# Performance of Blood-Based Indirect Scores Compared to Transient Elastography in Children with Chronic Liver Disease

**DOI:** 10.3390/diagnostics16071102

**Published:** 2026-04-06

**Authors:** Alexandru-Ștefan Niculae, Alina Grama, Monica Lupșor-Platon, Alexandra Mititelu, Gabriel Bența, Sorina Adam, Tudor Lucian Pop

**Affiliations:** 1 2nd Department of Pediatrics, Iuliu Hațieganu University of Medicine and Pharmacy, 3-5 Crișan Street, 400177 Cluj-Napoca, Romania; 2 Department of Radiology and Imaging, Iuliu Hațieganu University of Medicine and Pharmacy, 400012 Cluj-Napoca, Romania; 3 Department of Medical Imaging, “Prof. Dr. Octavian Fodor” Regional Institute of Gastroenterology and Hepatology, 400394 Cluj-Napoca, Romania

**Keywords:** non-invasive parameters scores, transient elastography, fibrosis, cirrhosis, children

## Abstract

**Background**: Chronic liver disease (CLD) in children requires long-term monitoring. Liver biopsy and transient elastography (TE) are resource-intensive methods that require specialized equipment and trained personnel. Simple indirect fibrosis scores based on routine laboratory parameters offer a potentially cost-effective alternative but have not been systematically evaluated in pediatric populations with diverse CLD etiologies. **Objectives**: This study aimed to assess the performance of several indirect fibrosis and cirrhosis scores in predicting significant (≥F2) and advanced (≥F3) fibrosis and cirrhosis (F4) in children with CLD using TE as a comparator. **Methods**: We retrospectively reviewed medical records of children with CLD evaluated at a tertiary center between January 2023 and June 2025. TE results and routine laboratory data were used to calculate fibrosis scores, including APRI, FIB-4, FibroIndex, FORNS, GPR, GUCI, King’s score, and Lok’s index. ROC analyses were performed to assess each score’s ability to discriminate significant fibrosis, advanced fibrosis and cirrhosis. Optimal cut-offs were established using the Youden index. **Results**: GPR showed the strongest concordance with TE-based fibrosis classification across both fibrosis thresholds, achieving an AUROC of 0.835 for significant fibrosis and a superior 0.917 for advanced fibrosis. FibroIndex and APRI also demonstrated good discriminatory power for advanced disease. Utilizing mathematically optimized cut-offs, GPR (0.45) and APRI (0.84) achieved good negative predictive values (100% and 95%) and sensitivities (100% and 85%) for advanced fibrosis, establishing them as potentially valuable screening tools. For cirrhosis detection (F4), Lok’s Index performed best (AUROC 0.854). **Conclusions:** In this diverse pediatric cohort, simple indirect scores—particularly GPR, APRI, and FibroIndex—demonstrated the highest concordance relative to TE findings, with negative predictive values up to 100% for GPR. This indicates that they can serve as reliable first-line screening tools when TE is unavailable. While their good negative predictive values allow for the confident exclusion of severe disease—potentially sparing many children from invasive testing—their low positive predictive values limit their role in definitive diagnosis. The systematic failure of adult-derived, age-dependent formulas in this cohort underscores the critical need for specialized pediatric biomarkers.

## 1. Introduction

Chronic liver diseases (CLDs) represent a leading cause of morbidity and mortality in society [1], with cirrhosis present only as the end stage on a spectrum of severity. However, healthcare needs expand before reaching this stage [2]. CLD in children represents a gradually changing landscape of diagnoses. Recent data indicate that the burden of chronic viral hepatitis is decreasing in children, but there are steady increases in other etiologies like metabolic-associated steatotic liver disease (MASLD, formerly known as non-alcoholic fatty liver disease or NAFLD). MASLD is projected to be the leading cause of CLD in children in the coming decades [3,4]. Accurate staging of liver fibrosis is the cornerstone of managing CLD, as it allows clinicians to plan and adjust therapeutic interventions. Identifying patients with advances fibrosis is particularly important in order to anticipate important potential complications such a portal hypertension, esophageal varices which are prone to bleeding, episodes of acute-on-chronic liver failure or the need for liver transplant [5]. That is why it is important to detect and evaluate patients with liver fibrosis at earlier stages and offer appropriate evidence-based care. Evaluating liver fibrosis has relied on liver biopsy, considered the gold standard of histological evaluation [3], but this is costly, time-consuming, and has significant associated risks and ethical limitations, especially in pediatric patients [6,7].

Over the past two decades, authors have proposed methods for evaluating liver fibrosis or cirrhosis using imaging and blood tests [8,9,10], with non-invasive methods gradually gaining more favor in clinical settings as tools for diagnosis and risk stratification in patients with CLD [11]. Imaging techniques include ultrasound-based methods, like transient elastography (TE) or shear wave elastography, as well as magnetic resonance elastography (MRE). The largest body of published scientific evidence is available for TE, while MRE is regarded as the more accurate imaging method for the non-invasive assessment of liver disease (NILDA) [12,13]. A systematic review addressed the issue of imaging-based NILDA compared to blood-based NILDA, and, in adults, imaging-based techniques are more reliable; a similar conclusion was found for children, although based on considerably less data. This highlights the need for continued studies in the pediatric population, although the authors concluded that imaging-based methods for assessing liver stiffness are likely as accurate in children as they are in adults [13]. Furthermore, recent guidelines have endorsed the use of TE for staging fibrosis in adult populations [14] and evidence from pediatric studies points to the fact that TE is accurate in detecting fibrosis compared to biopsy [15]. Blood tests that estimate liver fibrosis are broadly divided into direct tests, which include measurements of blood levels of biomarkers of fibrogenesis or of molecules associated with the degradation of the extracellular matrix [3,8], and indirect tests. Direct fibrosis marker tests include the enhanced liver fibrosis test (ELF), Hepascore, or FIBC3 scores [16]. They involve measuring components of the extracellular matrix, such as hyaluronic acid or components of collagen, and they are frequently more expensive. Indirect tests are represented by scores calculated from routinely available clinical and laboratory data. These scores were initially validated in adult populations, mostly in patients with chronic viral hepatitis. The most widely used are the aspartate aminotransferase-to-platelet ratio index (APRI) [17], Fibrosis-4 score (FIB-4) [18], FibroIndex [19], FORNS score [20], Göteborg University Cirrhosis Index (GUCI) [21], the King’ score [22] and Lok’s index [23]. Some research has evaluated the accuracy of these scores in adult populations with more diverse etiologies of CLD [9,24]. Scores such as FIB4 and APRI are associated with increased odds of detecting advancing stages of fibrosis in adults with viral hepatitides or MASLD [25]. Assessing liver fibrosis in children presents unique challenges that sets it apart from evaluating adults, mostly due to the different etiologies involved and other developmental factors. Some adult-derived scores take age into account, but these scores are inappropriate for evaluation in children. Furthermore, in pediatric cohorts, the spectrum of CLD is dominated by congenital, cholestatic or genetic/metabolic disorders, which is very different from the predominantly viral or lifestyle-related etiologies seen in adults.

Comparatively little research has been conducted to evaluate these scores in pediatric populations. One study from 2012 evaluated 68 children with CLD of varying etiologies who had undergone liver biopsy and found that APRI showed good performance for establishing the presence of advanced fibrosis, even superior to that of a patented test, Fibrotest^®^ [23]. More of this research focuses on children with MASLD [26,27]. A specific score has even been validated in comparison to liver histology for children with NAFLD, called the Pediatric NAFLD Fibrosis Score (PNFS) [28]. There are very little published data regarding the usefulness of these scores in children with CLD caused by anything other than MASLD, focusing mostly on cholestatic liver disease [6,29,30].

Non-invasive methods of quantifying liver fibrosis are considered accurate and cost-effective compared to liver biopsy [31]. There is increasing evidence that non-invasive methods for evaluating liver stiffness are appropriate for use in children, especially given their added comfort and safety for the patient [32,33]. In addition to these established tests, there is ongoing research into novel biomarkers that correlate with the degree of liver injury. For example, recent research has identified serum human epididymis protein 4 (HE4) and cytokeratin-18 (CK-18) as promising novel biomarkers for staging pediatric liver fibrosis. HE4, a protein typically involved in extracellular matrix accumulation, has been shown to be significantly elevated in children with CLD compared to healthy controls [34]. Similarly, CK-18 serves as a marker of hepatocyte injury and has been found to be significantly higher in pediatric CLD patients compared to healthy subjects [35].

Quantification of hepatic fibrosis using TE is well established, performs well compared with the gold standard of liver biopsy and histological evaluation, and is widely used to assess liver stiffness in children [6,36]. In adolescents with chronic hepatitis C virus infection, APRI and FIB-4 have been evaluated and correlate well with liver stiffness measurements (LSM) measured via TE [37]. It has been shown that LSM with TE can predict adverse health outcomes, both in relation to liver disease [5] and systemic complications [38]. Reference values for liver stiffness in children have recently been published [39]. TE by Fibroscan (Echosens, Paris, France) is a widely used method for assessing liver fibrosis [40].

While TE is a validated method for assessing liver stiffness and endorsed as an accurate non-invasive method for staging fibrosis, high capital costs and the need for specialized personnel limit its utility in resource-constrained settings or large-scale screenings. Simple, cost-effective biomarkers that stage liver fibrosis are therefore vital for stratifying CLD patients according to their clinical needs. Indirect blood-based tests for fibrosis scores offer a more accessible and cost-effective alternative. These scores rely on routine laboratory parameters already collected during standard patient workups, eliminating the need for additional procedures, equipment, or appointments. They can be easily calculated, interpreted without specialized expertise, and repeated over time to monitor disease progression or response to therapy. For settings where TE is unavailable or impractical, these simple scores offer a valuable first-line stratification tool to identify patients who may require closer monitoring or referral for further imaging. Moreover, they can be used to screen large populations, triage patients efficiently, and optimize resource allocation—all while maintaining a non-invasive approach that is particularly appropriate for pediatric populations. Expanding the validation and use of these scores beyond adult populations to children with chronic liver disease may therefore significantly improve access to early risk assessment and personalized care planning.

In this study, we aim to characterize the performance of these simple scores in evaluating liver fibrosis in a cohort of children with CLD of diverse etiologies. These findings will support the usefulness of these scores for clinicians who attend to children with a wide range of chronic liver disorders.

## 2. Materials and Methods

### 2.1. Participants

All patients included had an established diagnosis of CLD confirmed by their treating physician. Patients with acute hepatitis of any etiology, including acute viral infections or transient drug-induced liver injury, were not included in this cohort. Patients were enrolled retrospectively, with admissions for CLD follow-up between January 2023 and June 2025 being included. Using a chart review process, data on sex, age at evaluation, diagnosis, growth parameters, and laboratory and liver elastography were extracted from physical records. TE was performed by an experienced evaluator (MP) using FibroScan Expert 630 system (Echosens, Paris, France) with an M probe. TE and laboratory testing were performed on the same day whenever possible. In cases where this was not feasible due to routine clinical scheduling, only patients whose laboratory tests and TE were obtained within a maximum interval of seven days were included. Patients exceeding this interval were excluded from analysis to minimize potential temporal variability. Given the structural and slowly progressive nature of hepatic fibrosis, a significant stage change within this short interval was considered unlikely.

### 2.2. Variables

Where available, both the liver stiffness measured in kiloPascals (kPa) and the clinician’s interpretation of the results in METAVIR stages (F0 to F4) were recorded. According to the METAVIR staging, F0 corresponds to absent fibrosis, F1–F3 to progressively worsening liver fibrosis, and F4 to cirrhosis [29]. Patients were then grouped into two groups: 1. no or milder fibrosis (those for whom the chart review indicated fibrosis scores of F0 to F2 on TE) and 2. advanced fibrosis (those patients for whom the chart review indicated a fibrosis score of F3 or F4); thus, patients with advanced fibrosis represent a subset of those with significant fibrosis. Patients who presented with liver stiffness measurements corresponding to F2 or above were considered to have significant fibrosis [30]. Patients with F0 in our cohort were compared to previously published population standard reference values [28].

Data distribution was evaluated for normality using the Shapiro–Wilk test. Differences between the two groups were assessed using the non-parametric Mann–Whitney U test.

Children in our cohort who were classified as F0 and had a liver stiffness measurement in kPa documented in their chart were compared with the reference values reported in the literature [28] using a one-sample Wilcoxon rank-sum test. Indirect scores for quantifying fibrosis—APRI, FIB-4, FibroIndex, Forns, GPR, and liver cirrhosis, respectively—GUCI, King’s and Lok’s Index—were calculated, using previously published formulae [9,10,11,12,13,14,15,19]. These indirect scores rely on combinations of standard biochemical and hematological markers, including AST, ALT, GGT, platelet count, INR, albumin, serum IgG, cholesterol and age. The specific mathematical formulas and the required components for each score are detailed in Supplementary Table S1. Missing data were handled using available case analysis; patients were included in the ROC analysis for any specific score for which all required laboratory components were available, ensuring maximal use of the available retrospective data.

We assessed the effectiveness of these scores at classifying patients according to TE-defined fibrosis stages: 1. significant fibrosis (≥F2 METAVIR staging by TE); 2. advanced fibrosis (≥F3 on METAVIR staging by TE); or 3. the presence of cirrhosis (F4). This was evaluated using receiver operating characteristic (ROC) analysis and quantified as the area under the curve (AUROC) [31]. Optimal cut-offs for these use cases in our cohort were established using the Youden index. Also, cut-offs for their initial use cases, as described in the original publications for the investigated scores, were determined. Generally, an AUROC between 0.5 and 0.7 indicates poor test performance, between 0.7 and 0.9 indicates good performance, and above 0.9 indicates excellent performance [32]. However, recent studies evaluating the diagnostic performance of tests in pediatric hepatology have used more stringent criteria [3].

Statistical analysis was performed using Jamovi, version 2.6 [41].

## 3. Results

Between January 2023 and June 2025, 103 children (54 girls, 49 boys) were evaluated in our department for CLD and also had a valid LSM recorded in their medical charts. Both F0–F4 staging and liver stiffness (kPa) were available for 83 individual patients. CLD etiologies are summarized in Table 1. We classified the patients into major categories of CLD: a. chronic hepatitis (with a total of 35 patients), b. cholestatic liver disease (with a total of 38 patients), and c. metabolic and/or genetic liver disease (a total of 19 patients). Table 1 offers the different diagnoses that make up each category with the corresponding number of patients in each diagnosis subgroup summing up the total in that category. Demographic and clinical data are summarized in Table 2. Comparing the group with no or mild fibrosis to the group with advanced fibrosis shows significant differences. Those with more advanced fibrosis tend to be younger and predictably have altered laboratory parameters compared to those with no or mild fibrosis (Table 2; Supplementary Figure S1a–q provides bar plots for the comparisons in Table 2).

Forty-two patients with an LSM stage of F0 also had kPa values available in their medical records. Compared with published pediatric reference values, our participants did not show any significant differences (F0 patients n = 42, mean = 3.89 kPa, median = 4.15 kPa, standard deviation = 0.925, standard error = 0.143, compared to published reference standard of mean kPa 3.797 and SD = 0.4859, one-sample Wilcoxon ranked sum test, W 516, *p* = 0.42)

As expected, AST, ALT and GGT are elevated and the platelet count is lower in the advanced (F3–F4) fibrosis group compared to the no or milder fibrosis group (F0–F2). Scatterplots of kPa values and individual scores are available in Supplementary Figure S2a–i.

ROC analysis of the scores’ performances in discriminating the presence of significant and advanced fibrosis and cirrhosis, as well as Youden index-based cut-off criteria, is presented in Table 3 (ROC curves are available in Supplementary Figure S3a–c).

## 4. Discussion

Our study, for the first time, demonstrates the performance of several simple scores in detecting advanced hepatic fibrosis and cirrhosis in a diverse cohort of children with CLD. While it is not our aim to redefine the historical gold standard of liver biopsy, TE has become a well-regarded tool for evaluating liver fibrosis [11]. It has been established that well-performed TE is an acceptable alternative to liver biopsy for assessing fibrosis and its increasing use in clinical settings has been recommended by recent clinical practice guidelines that endorse its use for fibrosis detection and risk stratification [14]. There are several good reasons why TE is preferred to liver biopsy in children: a lack of pain or other organ injury, much higher acceptance from caregivers, and the ability to holistically evaluate a much larger area of tissue compared to the limited sample a biopsy offers. However, the availability of this procedure is generally restricted to tertiary centers, especially in resource-limited settings, requiring patients to travel and limiting its use compared to that of easily available blood tests. Although liver fibrosis is the main determinant of liver stiffness, which is the physical characteristic of the tissue that allows elastography to be performed, other factors also influence stiffness. Food intake, inflammation and cholestasis can all influence LSM via elastography [42,43].

Previous studies with similar methodologies have focused exclusively on children with MASLD [19,36,37,38]. In children, MASLD, APRI, FIB-4 and PNFS perform poorly in discriminating fibrosis stages compared to the gold standard of histological assessment and this highlights the need for well-established staging tests tailored to pediatric patients [26].

To our knowledge, no previous study has systematically examined the performance of these scores in a pediatric cohort with a diverse range of CLD causes. Most of these simple scores were developed in adult cohorts with viral hepatitis C or B, but published studies have assessed some of these scores in more diverse etiologies of CLD in adults, such as autoimmune hepatitis [39] or Wilson’s disease [40].

In our subset analysis, 42 patients with an LSM stage of F0 also had corresponding kPa values documented in their medical records. Compared with published pediatric reference values, this group did not exhibit any significant differences in liver stiffness. These patients, classified as having no detectable fibrosis (F0) based on TE, showed kPa values that closely align with normative data [28]. This finding is clinically relevant for several reasons. First, it provides internal validation of TE use in our pediatric population by showing that LSM values in patients with F0 staging fall within expected reference ranges. This adds confidence in the classification of fibrosis stages using TE in children, especially in clinical or research settings where liver biopsy is not feasible. Second, it reinforces the reliability of using non-invasive methods such as TE to monitor liver health longitudinally. Finally, by confirming that our F0 group mirrors healthy reference populations, it strengthens the interpretability of elevated LSM values in other patients within the cohort, highlighting TE’s discriminative value in detecting early liver pathology.

Our cohort contains children with viral hepatitides, autoimmune hepatitis, cholestatic liver disease and metabolic liver disease (such as Wilson’s disease and glycogen storage diseases). A breakdown of the etiologies of CLD in our cohort by age group and fibrosis stage is provided in Supplementary Figure S4.

The simple scores, all of which rely on readily available laboratory values, can be of value to clinicians who monitor these children for many years and who must prepare them for a successful transition to adult care. Predicting the presence of significant or advanced fibrosis based on these scores or monitoring adherence to therapy and evaluating the reduction or progression of fibrosis more frequently will help identify patients who need frequent specialist evaluation, as opposed to those who can be monitored with relative confidence by their primary care provider (PCP) [41]. Moreover, once the presence of advanced fibrosis is suspected, PCPs or specialists can more readily establish the need for interdisciplinary consultations and evaluations for children with CLD.

From the outset, some of the scores developed in adults have very specific limitations when translated into pediatric research. Scores that include age (FIB4, FORNS, King’s score) as a parameter in their formula performed poorly compared to some of the others.

In the original paper describing the FIB-4 score, the authors also concluded that age contributed significantly to the overall results. In analyzing patients with discrepancies between histologically determined fibrosis grade and scores derived from the newly proposed score, the authors found that older patients had inappropriately high scores and were more likely to be misclassified as having more severe fibrosis. Conversely, the authors indicated that young age would falsely lower scores and lead to misclassification of patients as having less severe fibrosis than they actually have. Given the poor performance of the FIB-4 score in our cohort (mean age 9.17 years), the prediction of the original authors is clearly reflected in our study.

The Forns score also includes age as a parameter. It is a test that, like others discussed here, was developed in adult cohorts of patients with hepatitis C. Given the way age is incorporated into the formula, most scores resulted in negative values (while mathematically valid, it lacks any intuitiveness preferable for an accessible tool). We do not consider the formula appropriate for pediatric use.

The AST-to-ALT ratio also performed poorly in our cohort, not surprisingly given the diverse range of diagnoses within our cohort, from cholestatic to metabolic to viral and autoimmune diseases. This probably creates overlapping biochemical patterns that ultimately obscure this ratio’s ability to discriminate between stages of fibrosis, likely since is simply relies on the two transaminases.

### 4.1. Discrimination of Significant (F2 and Above) and Advanced (F3–F4) Versus No or Milder Form, as Determined by TE

The AST-to-ALT ratio, APRI, FibroIndex, GPR, FIB-4, Forns, and King’s score were individually compared with TE regarding discrimination between non-advanced and advanced fibrosis. In our cohort, patients in the advanced fibrosis group also showed statistically significant increases in AST, ALT, GGT, and INR and statistically significant decreases in albumin and platelets (Table 2 and Supplementary Figure S1a–q).

The best-performing score for discriminating between milder and more severe liver fibrosis relative to TE was GPR, with good results also obtained with APRI and FibroIndex. All these scores use laboratory measurements that were clearly significantly different between the two groups in our cohort. There is one caveat regarding serum IgG levels: 14 of the 103 patients with completed TE measurements lacked IgG levels measured at a sufficiently close time point, so FibroIndex could not be calculated for these patients and they were subsequently excluded from the ROC analysis of this score. This might also point towards a limitation in considering the use of FibroIndex in primary care settings, since measuring immunoglobulin levels might not be easily or rapidly available in any community or in low-resource settings.

GPR was the most robust performer for both thresholds evaluated. For detecting advanced fibrosis (F3 and above), an optimized cut-off of 0.45 achieved 100% sensitivity and 100% NPV, indicating that every child with advanced fibrosis in this cohort was correctly identified, and a negative result at this threshold was completely reliable for ruling out advanced fibrosis. A higher cut-off of 0.78 was identified for detecting significant fibrosis (F2 and above), and while this might seem counterintuitive, it is a result of the Youden index method for identifying this cut-off. The resulting specificity (90.67%) and the resulting NPV (at 88.31%) remained high, making it a reliable tool for excluding significant fibrosis in a clinical setting.

APRI is also a robust candidate for screening due to its simplicity and the reliability it demonstrated for advanced disease stages in our cohort. For detecting both significant (≥F2) and advanced (≥F3) fibrosis, we identified the same optimized cut-off of ≥0.84. This threshold yielded a sensitivity of 85% and an NPV of 95.6% for detecting advanced fibrosis. The high NPV for both APRI and GPR suggests that these non-invasive markers can effectively identify children at low risk of advanced fibrosis, potentially deferring the need for more invasive diagnostic interventions.

However, the fact that these scores show the same threshold for both classification tasks in the case of APRI and a lower threshold for GPR in the classification task for advanced fibrosis warrants further discussion. Since liver function tests are expected to worsen as disease progresses, one would expect higher thresholds for detecting more advanced as compared to milder fibrosis. Yet, in our cohort, there is non-linear progression in liver function tests as disease worsens (see Supplementary Material S5—table and boxplots). Both mean and median GGT levels peak in patients with F3 fibrosis and drop by more than 50% in patients with F4 fibrosis. Median AST levels show only a minor difference (a small 6% drop from F3 to F4) in our cohort. GGT and AST are included in the numerators of their respective scores (GPR and APRI). Their relative decline as fibrosis stage increases likely outpaces that of platelets. In our cohort, the largest drop in the median platelet count is 34%, seen from F2 to F3, while the drop from F3 to F4 is only about 20%. This observation might point to the fact that in our cohort, F4 patients tend to present with what might be interpreted as a “burnt-out” biochemical profile, when liver function tests are lower compared to patients with less advanced disease due to parenchymal loss caused by the progression of fibrosis. Moreover, in our cohort, F2 patients represented the smallest group (n = 7, see Supplementary Figure S4) and such a low number within this subgroup likely makes it difficult to capture the true biochemical profile of this subpopulation. In our group of patients, the F2 subgroup shows median values for AST, GGT and platelets that are very similar to those of F0/1 patients; thus, their biochemical profiles and scores are not significantly different from those of neighboring stages (see Supplementary Material S5).

Similarly, FibroIndex has good diagnostic accuracy for identifying both significant (≥F2) and advanced (≥F3) fibrosis in our pediatric patients using the same threshold (≥1.46). This is to be expected as there are no significant differences across any pair of subgroups for IgG levels in our cohort and no differences between any pair of subgroups within the significant fibrosis group (F2–4). However, its clinical application is limited by the specific requirement for IgG levels. When applied to the detection of advanced fibrosis, a cut-off of 1.46 achieved a sensitivity of 73.68% and an NPV of 92.19%. Interestingly, maintaining the same threshold of 1.46 for significant fibrosis resulted in a high NPV of 86.94% paired with a robust specificity of 91.67%. These results highlight the potential of FibroIndex as a highly accurate marker, provided that the necessary laboratory components are available at the time of clinical assessment.

### 4.2. Discrimination of Cirrhosis (F4) Versus All Other Stages of Liver Involvement (F0–F3), as Determined by TE

The analysis of performance in discriminating cirrhosis was applied only to scores developed specifically for this purpose in their original publications: King’s score [16], Lok’s odds Index [17], and GUCI [13]. As with the previous score, these three scores were originally developed on adult cohorts with viral hepatitis.

King’s score achieved an AUROC of 0.733, and it is less reliable than the others, most likely because age is a parameter.

Lok’s odds Index and the GUCI score show good performance. None of these scores require age as a factor; however, in our cohort, four observations had to be dropped from the ROC analysis of these scores because they lacked an INR value within one week of TE evaluation. Both scores require INR values for calculation. Lok’s odds Index demonstrated the strongest performance for excluding cirrhosis. At an optimized cut-off of 0.34, it achieved a sensitivity of 92.3% and a remarkably high NPV of 98.55%. From a clinical standpoint, this is quite meaningful: if a child’s score falls below 0.34, a clinician can be nearly 99% certain that cirrhosis is not present, potentially deferring more invasive confirmatory testing. The NLR of 0.1 (0.0972, rounded to two decimal places) is the only one of our cirrhosis-specific scores that satisfies the “strong rule-out” threshold of <0.1, reinforcing its utility as a reliable first-line filter.

### 4.3. Strengths and Limitations

We chose to include only children who had TE performed in very close proximity to laboratory blood analyses to reduce confounding factors related to potential changes in liver stiffness over time, due to either disease progression or the therapeutic effects of medication.

We have evaluated these scores in a pediatric cohort with a wide range of CLD causes, providing additional robustness to their accuracy. To date, this is the only study to evaluate such a large number of scores in a pediatric cohort. However, our study is based on a single center’s experience. Moreover, other significant causes of CLD in children, such as secondary liver damage in cystic fibrosis, are not represented in our cohort.

Our data suggest that these scores find their greatest value as screening—or “rule-out”—tools. By utilizing cut-offs optimized to maximize the Youden index, we found that GPR (at 0.45) and APRI (at 0.84) provided exceptional NPVs for advanced fibrosis of 100% and 95.65%, respectively. With such high sensitivity—100% for GPR—a negative result can be viewed with confidence, potentially sparing many children from the risks of a liver biopsy. However, the diagnostic “rule-in” power of these tests remains their primary weakness. The PPV of GPR was only 50%, while that of APRI was 52.9%. This indicates that half of the “positive” results at these thresholds are false positives.

Our study presents several strengths that we consider important to highlight here. First, all TE measurements were performed by a single, highly skilled expert, thus eliminating a source of confounding variability. The fact that patients with F0 staging in our cohort do not differ from published normative data further confirms the validity of the TE measurements. Second, our cohort, by design, includes a diverse array of etiologies, as patients were recruited from our department. However, the inclusion of diverse etiologies increases heterogeneity and may introduce biochemical variability that could influence score performance. Disease-specific differences in laboratory patterns may partially confound the discriminatory ability of these indices. Clinically, patients present specific, distinct etiologies, and the laboratory patterns associated with conditions such as biliary atresia can differ from those seen in metabolic or autoimmune diseases. This diversity potentially introduces confounding biochemical variables that may obscure the discriminatory power of these scores. However, we maintain that this design reflects the ‘real-world’ screening environment, where a robust triage tool must be effective across a wide spectrum of disorders. The high NPVs observed for GPR and APRI in this varied population suggest that these markers function as dependable screening tools in the undifferentiated clinical setting. Future multi-center studies should try to confirm these results in larger, disease-specific subgroups to help fine-tune the diagnostic thresholds even more.

Our study has limitations typical of retrospective studies. Since data were gathered as a chart review of presentations of children with CLD in a tertiary center, it is reasonable to assume that children with more severe liver disease are overrepresented. Moreover, some patients did not have a TE examination documented in their health record, had incomplete information about the TE examination, or had the TE examination more than one week before or after the blood samples. We were subsequently unable to include them in the ROC analyses of the scores. Among children who had blood drawn near the time of TE, some had missing values for certain parameters, such as INR and IgG.

In addition, our dataset is characterized by both class imbalance, with a substantially larger F0–F2 group (n = 82) compared to F3–F4 (n = 21), and overlap in score distributions across fibrosis stages, involving F2 patients. These factors are known to influence ROC-derived thresholds and can result in non-monotonic cut-off values when optimizing sensitivity and specificity using the Youden index.

Furthermore, the duration of CLD in a pediatric context warrants specific consideration. In our cohort, a substantial proportion of patients were affected by congenital or early-onset conditions such as biliary atresia, genetic/metabolic disorders, or vertically transmitted viral infections. In these settings, liver involvement typically begins in infancy or early childhood, and therefore chronological age may approximate cumulative disease duration. However, we acknowledge that this relationship is not uniform across all etiologies. Variability in the timing of diagnosis, disease progression rates, and therapeutic interventions may introduce heterogeneity that a retrospective design cannot fully capture. For this reason, age should be interpreted as an indirect and approximate surrogate for disease duration rather than an exact measure. Prospective longitudinal studies would be better suited to precisely characterize the impact of disease duration on fibrosis progression in pediatric populations

While we acknowledge that the velocity of fibrosis progression—specifically whether advanced stages (F3–F4) are reached rapidly or over a more protracted course—is a vital clinical and prognostic metric, characterizing these dynamics was outside the scope of our current retrospective methodology. Our study was designed to evaluate the immediate diagnostic accuracy of these scores as screening tools at a single clinical time point. The nuances of progression rates, and the longitudinal impact of specific therapies are better addressed through prospective study designs, which remain a necessary direction for future pediatric research.

While liver biopsy remains the historical gold standard, its routine use in pediatrics is limited by ethical considerations and parental refusal. We use TE as our primary reference, as it is a clinically accepted, non-invasive alternative for assessing pediatric liver stiffness, which correlates very well histopathology results [43]. There are certain limitations when it comes specifically to TE. While for our study a single expert evaluated the participants, thus reducing the confounding factors, it is reasonable to assume that TE presents inter-operator variability when multiple operators perform this evaluation. Furthermore, distinguishing between adjacent intermediate scores can be nuanced and challenging. Future prospective studies should take this into account and address this issue by utilizing standardized acquisition protocols in larger, well-stratified cohorts.

Ultimately, while GPR and, to a lesser extent, APRI are promising filters, no single score in our study exhibited sufficiently good performance to serve as a standalone replacement for TE or biopsy. This gap in diagnostic precision highlights the need for dedicated pediatric biomarkers that account for the unique physiological and laboratory characteristics of children.

The findings in our study have highlighted several key areas for future research in pediatric hepatology. Future research should focus on biomarkers and thresholds that are inherently calibrated to the physiological and laboratory characteristics of children, moving away from adult models that introduce systematic age-based bias. The poor performance of some adult-derived scores, especially age-dependent scores, underscores the need to develop dedicated pediatric fibrosis scores. To begin with, these scores can be estimated using retrospective cohorts and subsequently validated in prospective, longitudinal cohorts.

Our results indicate that while standalone indirect scores are valuable screening filters, they currently lack the positive predictive power for definitive diagnosis. Efforts should focus on developing multimodal prediction models that integrate clinical data, laboratory parameters, and TE measurements. Combining the high negative predictive value of biochemical scores like GPR and APRI with the liver stiffness data provided by TE could be one way to bridge the current gap in diagnostic precision.

## 5. Conclusions

In conclusion, simple scores derived from routine clinical values are valuable tools for both primary care providers and specialists managing pediatric CLD. In this study, GPR (AUC 0.917), APRI (AUC 0.857), and FibroIndex (AUC 0.845) demonstrated the highest accuracy for discriminating advanced fibrosis (≥F3), compared to the clinically accepted comparator of TE. For the specific identification of cirrhosis (F4), Lok’s odds Index was the most effective score (AUC 0.854). Our results indicate that the primary clinical utility lies in their high NPVs to exclude advanced disease; their relatively low PPVs indicate that they should not be used for definitive diagnosis. Further multi-center, longitudinal studies will be essential to evaluate the performance and stability of these scores over time and to determine whether they can be fully integrated into routine pediatric practice.

## Figures and Tables

**Table 1:** Etiologies of CLD in study cohort.

Frequency of Etiologies of CLD in Our Cohort	
	N 103
**Cholestatic liver disease**	**38**
Biliary atresia	29
Alagille syndrome	4
PFIC	2
Congenital malformation of the biliary tract, other than BA	3
**Chronic hepatitis**	**35**
Hepatitis B	9
Hepatitis C	1
Autoimmune hepatitides	24
Giant cell hepatitis	1
**Metabolic or genetic liver disease**	**19**
Wilson’s disease	8
Glycogen storage disease	5
Hereditary fructose intolerance	4
Others	2
**Chronic liver disease, nos**	**11**

nos—not otherwise specified, PFIC—progressive familial intrahepatic cholestasis, BA—biliary atresia.

**Table 2:** Clinical parameters and differences across groups for patients with CLD.

Variable	F0–F2 (N = 82) Median [IQR]	F3–F4 (N = 21) Median [IQR]	*p*-Value *
**Age (months)**	112.50 [82.75]	52.00 [106.00]	0.038
**Weight (kg)**	31.50 [24.98]	18.00 [27.50]	0.065
**Height (cm)**	132.50 [44.75]	115.00 [61.00]	0.21
**WBC (×10^9^/L)**	7.44 [3.39]	8.63 [7.30]	0.232
**RBC (×10^12^/L)**	4.73 [0.60]	4.20 [0.98]	<0.001
**Hemoglobin (g/dL)**	12.90 [1.98]	11.40 [3.40]	0.009
**Platelets (×10^9^/L)**	265.0 [108.0]	132.0 [98.0]	<0.001
**INR**	0.97 [0.11]	1.01 [0.32]	0.006
**AST (U/L)**	40.50 [48.13]	111.0 [156.0]	<0.001
**ALT (U/L)**	37.00 [72.25]	94.00 [168.4]	0.002
**GGT (U/L)**	25.25 [47.75]	99.0 [192.0]	<0.001
**Total Bilirubin (mg/dL)**	0.54 [0.32]	1.28 [3.52]	<0.001
**Conjugated Bilirubin (mg/dL)**	0.18 [0.08]	0.95 [1.83]	<0.001
**Serum Cholesterol (mg/dL)**	163.0 [43.0]	152.0 [125.5]	0.681
**Triglycerides (mg/dL)**	70.0 [66.0]	107.0 [62.0]	0.069
**Serum IgG (mg/dL)**	1239.3 [552.9]	1391.0 [914.8]	0.323
**Albumin (g/dL)**	4.70 [0.40]	3.77 [0.90]	<0.001

* Mann–Whitney U test for differences across groups.

**Table 3:** (**a**) Diagnostic accuracy parameters and cut-off values for detecting significant (≥F2) and advanced (≥F3) fibrosis. (**b**) Diagnostic accuracy parameters and cut-off values for detecting cirrhosis.

**(a)**			
**Score and Metric**	**Significant Fibrosis (≥F2)**	**Advanced Fibrosis (≥F3)**	**Original Thresholds (Literature)**
**AST/ALT (n = 103)**			
**Cut-off**	>1.04	≥1.83	-
**Se/Sp (%)**	50.00/56.00	28.57/89.02	
**PPV/NPV (%)**	29.79/75.00	40.00/82.95	
**PLR/NLR**	1.14/0.89	2.60/0.80	
**AUROC [95% CI] (*p*)**	0.476 [0.349–0.604] (0.716)	0.570 [0.429–0.712] (0.331)	
**Overall Accuracy (%)**	64.22	76.7	
**APRI (n = 103)**			
**Cut-off**	≥0.84	≥0.84	≥1.5 to rule in F2 or above
**Se/Sp (%)**	71.43/81.33	85.71/80.49	
**PPV/NPV (%)**	58.82/88.41	52.94/95.65	
**PLR/NLR**	3.83/0.35	4.39/0.18	
**AUROC [95% CI] (*p*)**	0.757 [0.631–0.882] (<0.001)	0.857 [0.759–0.954] (<0.001)	
**Overall Accuracy (%)**	78.64	81.55	
**FibroIndex** **(n = 85)**			
**Cut-off**	≥1.46	≥1.46	≥2.25 to rule in F2 or above
**Se/Sp (%)**	64.00/91.67	73.68/89.39	
**PPV/NPV (%)**	76.19/85.94	66.67/92.19	
**PLR/NLR**	7.68/0.39	6.95/0.29	
**AUROC [95% CI] (*p*)**	0.791 [0.663–0.918] (<0.001)	0.845 [0.719–0.971] (<0.001)	
**Overall Accuracy (%)**	83.53	85.88	
**FORNS (n = 88)**			
**Cut-off**	≥−22.19	≤−26.15	<4.2 to rule out F2 or above
**Se/Sp (%)**	50.00/79.03	47.37/81.16	
**PPV/NPV (%)**	50.00/79.03	40.91/84.85	
**PLR/NLR**	2.39/0.63	2.51/0.65	
**AUROC [95% CI] (*p*)**	0.569 [0.408–0.730] (0.401)	0.515 [0.325–0.705] (0.878)	
**Overall Accuracy (%)**	70.45	73.86	
**FIB-4 (n = 103)**			
**Cut-off**	≥0.39	≥0.55	<1.45 (rule out F3)
**Se/Sp (%)**	46.43/90.67	42.86/97.56	>3.25 (rule in F3)
**PPV/NPV (%)**	65.00/81.93	81.82/86.96	
**PLR/NLR**	4.97/0.59	17.57/0.59	
**AUROC [95% CI] (*p*)**	0.631 [0.483–0.780] (0.083)	0.607 [0.429–0.784] (0.238)	
**Overall Accuracy (%)**	78.64	86.41	
**King’s (n = 99)**			
**Cut-off**	≥1.42	≥2.36	>12.3 to rule in F3 or above
**Se/Sp (%)**	75.00/57.75	61.90/79.49	
**PPV/NPV (%)**	41.18/85.42	44.83/88.57	
**PLR/NLR**	1.78/0.43	3.02/0.48	
**AUROC [95% CI] (*p*)**	0.691 [0.567–0.815] (0.003)	0.725 [0.584–0.867] (0.002)	
**Overall Accuracy (%)**	62.63	75.76	
**GPR (n = 103)**			
**Cut-off**	≥0.78	≥0.45	>0.32 to rule in F3 or above
**Se/Sp (%)**	67.86/90.67	100.00/74.39	
**PPV/NPV (%)**	73.08/88.31	50.00/100.00	
**PLR/NLR**	7.27/0.36	3.90/0.00	
**AUROC [95% CI] (*p*)**	0.835 [0.744–0.926] (<0.001)	0.917 [0.864–0.970] (<0.001)	
**Overall Accuracy (%)**	84.47	79.61	
**(b)**			
**Score & Metric**	**Optimized Cut-Off**	**Performance Metrics**	**Original Thresholds (Literature)**
**Lok’s odds Index** **(n = 99)**	≥0.34		>0.5 (to rule in cirrhosis)
**Se/Sp (%)**		92.31/79.07	
**PPV/NPV (%)**		40.00/98.55	
**PLR/NLR**		4.41/0.10	
**AUROC [95% CI] (*p*)**		0.854 [0.705–1.00] (<0.001)	
**Overall Accuracy (%)**		80.81	
**King’s Score** **(n = 99)**	≥2.52		>16.7 (to rule in cirrhosis)
**Se/Sp (%)**		69.23/80.23	
**PPV/NPV (%)**		34.62/94.52	
**PLR/NLR**		3.50/0.17	
**AUROC [95% CI] (*p*)**		0.733 [0.548–0.917] (0.014)	
**Overall Accuracy (%)**		78.79	
**GUCI (n = 99)**	≥0.86		<1 (to rule out cirrhosis)
**Se/Sp (%)**		84.62/76.74	>4 (to confirm cirrhosis)
**PPV/NPV (%)**		35.48/97.06	
**PLR/NLR**		3.64/0.20	
**AUROC [95% CI] (*p*)**		0.828 [0.685–0.972] (<0.001)	
**Overall Accuracy (%)**		77.78	

Se—sensitivity; Se—specificity; PPV/NPV—positive/negative predictive value; PLR/NLR—positive/negative likelihood ratio; AUROC—area under receiver operating characteristics analysis; patients with advanced (≥F3) fibrosis represent a subset of those with significant (≥F2) fibrosis.

## Data Availability

Curated data and Jamovi files are available upon reasonable request from the authors after an appropriate amount of time after publication.

## Supplementary Materials

The following supporting information can be downloaded at: https://www.mdpi.com/article/10.3390/diagnostics16071102/s1, Figures S1: a–q for parameters compared between groups (F0–2 versus F3–4 groups); Figures S2: a–i scatterplots, Figures S3: a–c ROC Curves; Figure S4: bar charts of etiologies and fibrosis staging across age groups; Figure S5: Descriptive statistics of parameters and scores for APRI GPR FibroIndex

## Abbreviations

The following abbreviations are used in this manuscript:

CLD	Chronic liver disease
TE	Transient elastography
APRI	AST-to-platelet ratio index
GPR	Gamma glutamyl transferase-to-platelet ratio
INR	Internation normalized ratio
IgG	Immunoglobulin G
kPa	kiloPascal
Se/Sp	Sensitivity/Specificity
PPV/NPV	Positive predictive value/negative predictive value
LR+/LR−	Positive/negative likelihood ratio
ROC	Receiver operating characteristics
AUC	Area under the curve
AST	Aspartate amino transferase
ALT	Alanine amino transferase
GGT	Gamma glutamyl transferase
WBC	White blood cell
RBC	Red blood cell
Hb	Hemoglobin
LSM	Liver stiffness measurement
HE4	serum human epididymis protein 4
CK18	cytokeratin-18
