# Supplementary material for: Performance of Blood-Based Indirect Scores Compared to Transient Elastography in Children with Chronic Liver Disease
Source: Diagnostics (Basel). 2026 Apr 6;16(7):1102. doi: 10.3390/diagnostics16071102 (PMC13074151; doi:10.3390/diagnostics16071102)
Supplement: Supplementary file 1 [file diagnostics-16-01102-s001.zip › Supplementary Material S5 Descriptive statistics of parameters and scores for APRI GPR Fibroindex.pdf]

Supplementary Material S5

Descriptive statistics of parameters and scores for APRI GPR Fibroindex

Descriptives

							Shapiro-Wilk	
	FIBROSCAN	N	Mean	Median	SD	IQR	W	p
AST	F0	51	90.306	42.800	123.595	48.650	0.583	<.001
	F1	24	59.254	39.000	54.695	36.275	0.594	<.001
	F2	7	87.571	29.000	109.119	88.000	0.684	0.002
	F3	8	145.137	118.000	103.094	161.500	0.915	0.388
	F4	13	219.685	111.000	321.498	92.000	0.573	<.001
GGT	F0	51	48.741	24.000	55.723	45.500	0.645	<.001
	F1	24	97.150	27.950	178.687	37.425	0.516	<.001
	F2	7	114.000	33.000	198.483	56.000	0.576	<.001
	F3	8	365.538	197.500	377.704	457.750	0.851	0.098
	F4	13	187.738	93.000	287.959	97.000	0.538	<.001
Platelets	F0	51	310.686	288	95.250	101.500	0.881	<.001
	F1	24	262.792	247.500	95.404	67.500	0.885	0.010
	F2	7	255.000	252	86.583	114.000	0.983	0.972
	F3	8	200.750	166.000	137.046	166.750	0.909	0.345
	F4	13	189.308	132	170.924	76.000	0.681	<.001
APRI	F0	51	0.896	0.382	1.319	0.504	0.554	<.001
	F1	24	0.588	0.467	0.379	0.268	0.681	<.001
	F2	7	1.192	0.393	1.573	1.364	0.690	0.003
	F3	8	2.875	1.908	2.787	1.234	0.725	0.004
	F4	13	4.490	2.254	6.193	4.856	0.664	<.001
GPR	F0	51	0.315	0.182	0.319	0.302	0.710	<.001
	F1	24	0.859	0.286	1.449	0.380	0.553	<.001
	F2	7	0.930	0.179	1.559	0.617	0.608	<.001
	F3	8	6.265	2.245	10.833	3.523	0.552	<.001
	F4	13	2.187	1.529	2.067	1.612	0.778	0.004
IgG	F0	42	1245.221	1236.300	478.543	577.000	0.959	0.140

	F1	21	1241.119	1225.700	307.448	338.400	0.942	0.238
	F2	6	1326.150	1374.100	693.487	469.875	0.972	0.907
	F3	8	1743.263	1495.050	1175.311	1312.675	0.900	0.286
	F4	12	1375.383	1390.950	609.649	765.625	0.978	0.972
Albumin	F0	47	4.733	4.800	0.255	0.300	0.957	0.083
	F1	22	4.568	4.675	0.349	0.387	0.941	0.209
	F2	7	4.657	4.700	0.458	0.300	0.935	0.593
	F3	8	4.037	3.850	0.573	1.050	0.862	0.125
	F4	12	3.701	3.720	0.626	0.875	0.923	0.311
FibroIndex	F0	40	0.663	0.731	0.628	0.722	0.958	0.139
	F1	20	1.078	1.079	0.390	0.554	0.971	0.766
	F2	6	1.166	0.948	0.996	0.706	0.922	0.520
	F3	8	1.986	1.706	0.829	1.300	0.908	0.340
	F4	11	2.267	2.250	2.354	1.332	0.882	0.110

Plots

AST

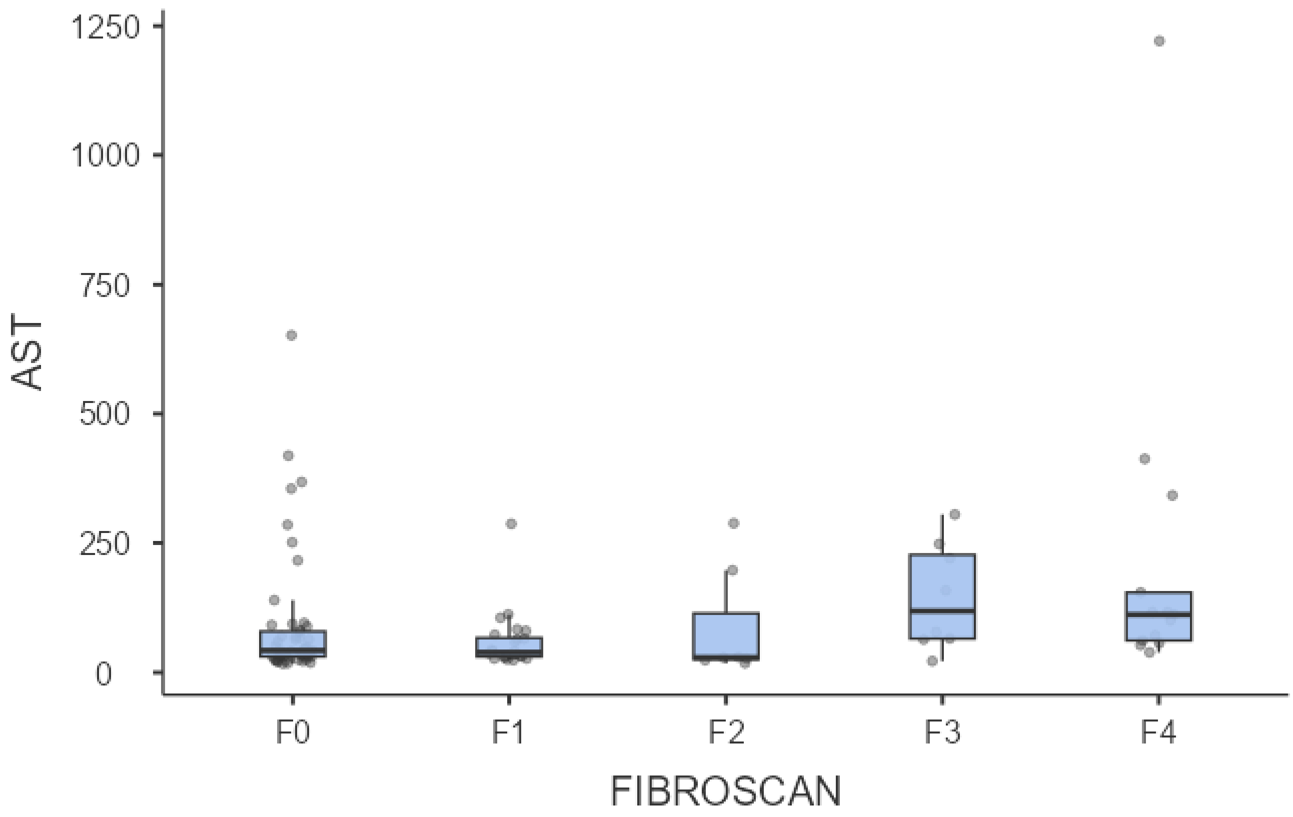

GGT

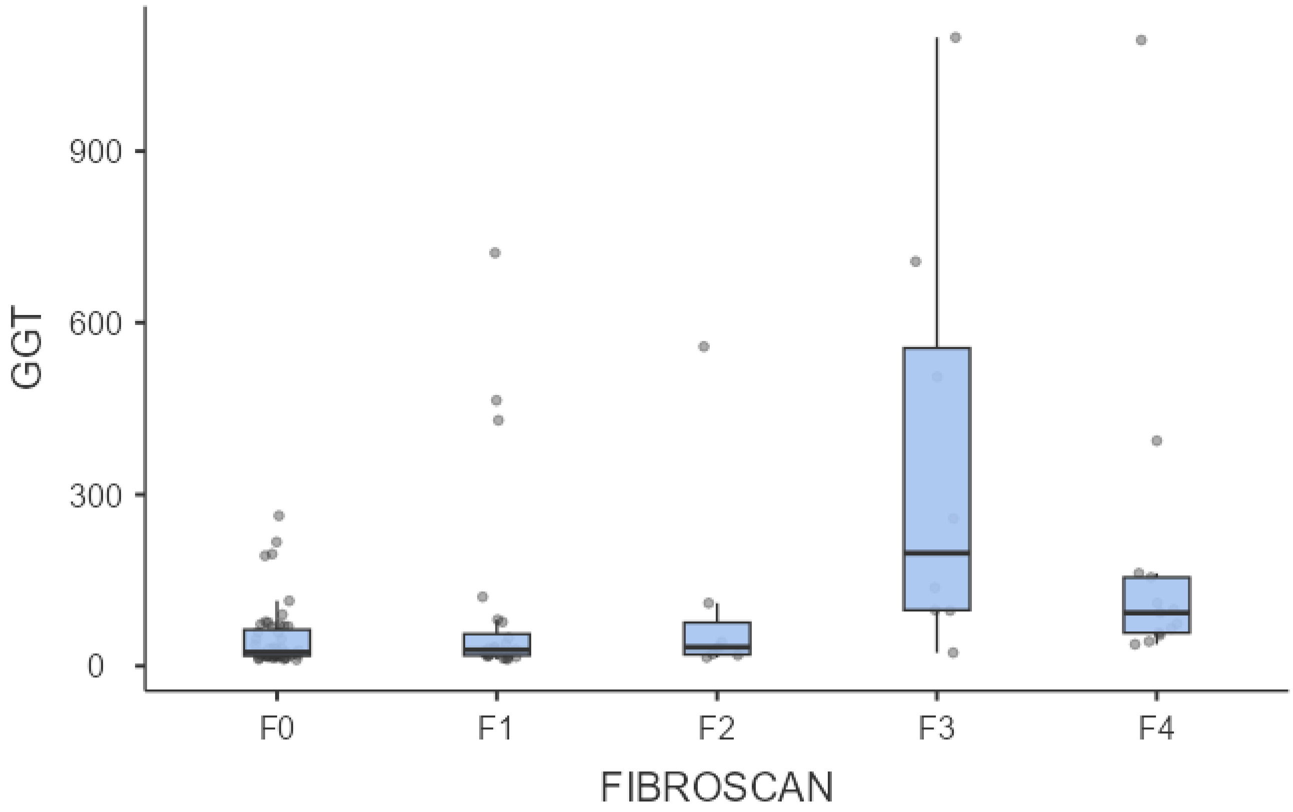

Platelets

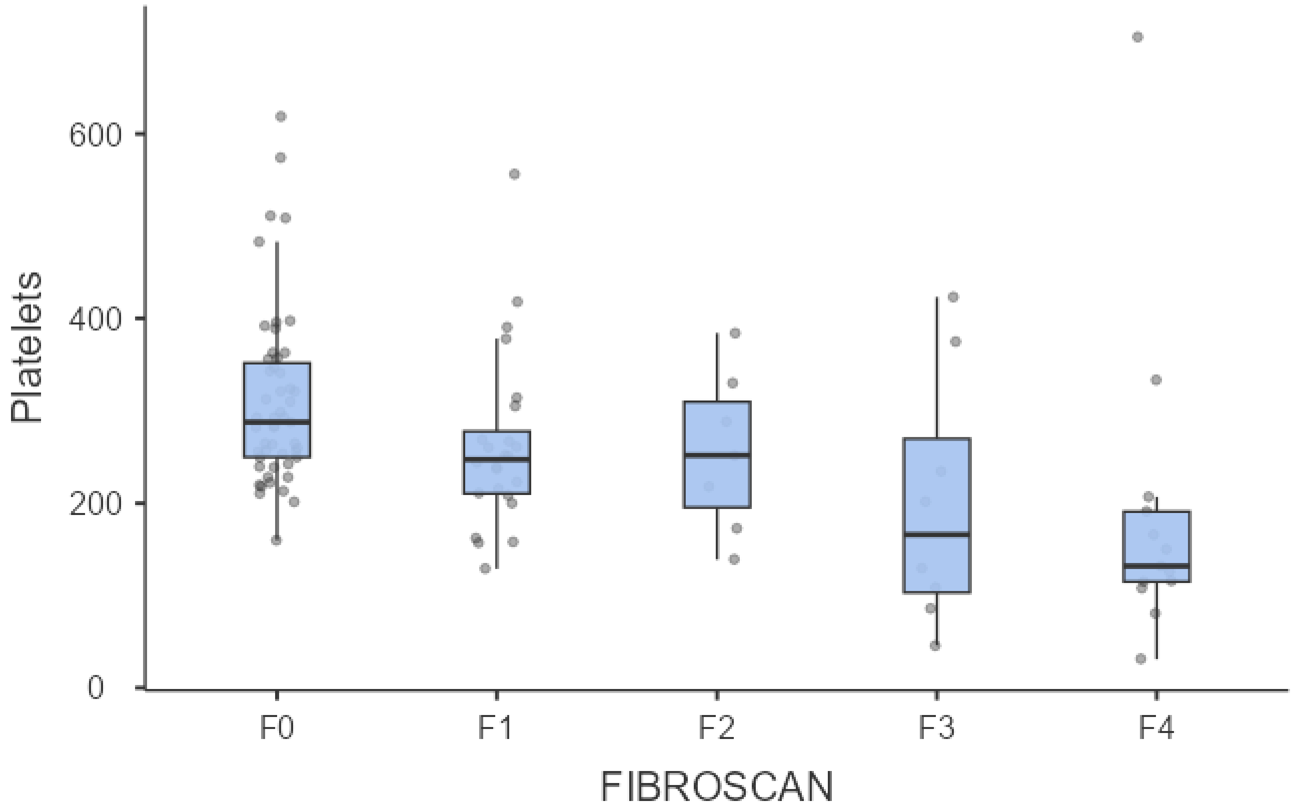

APRI

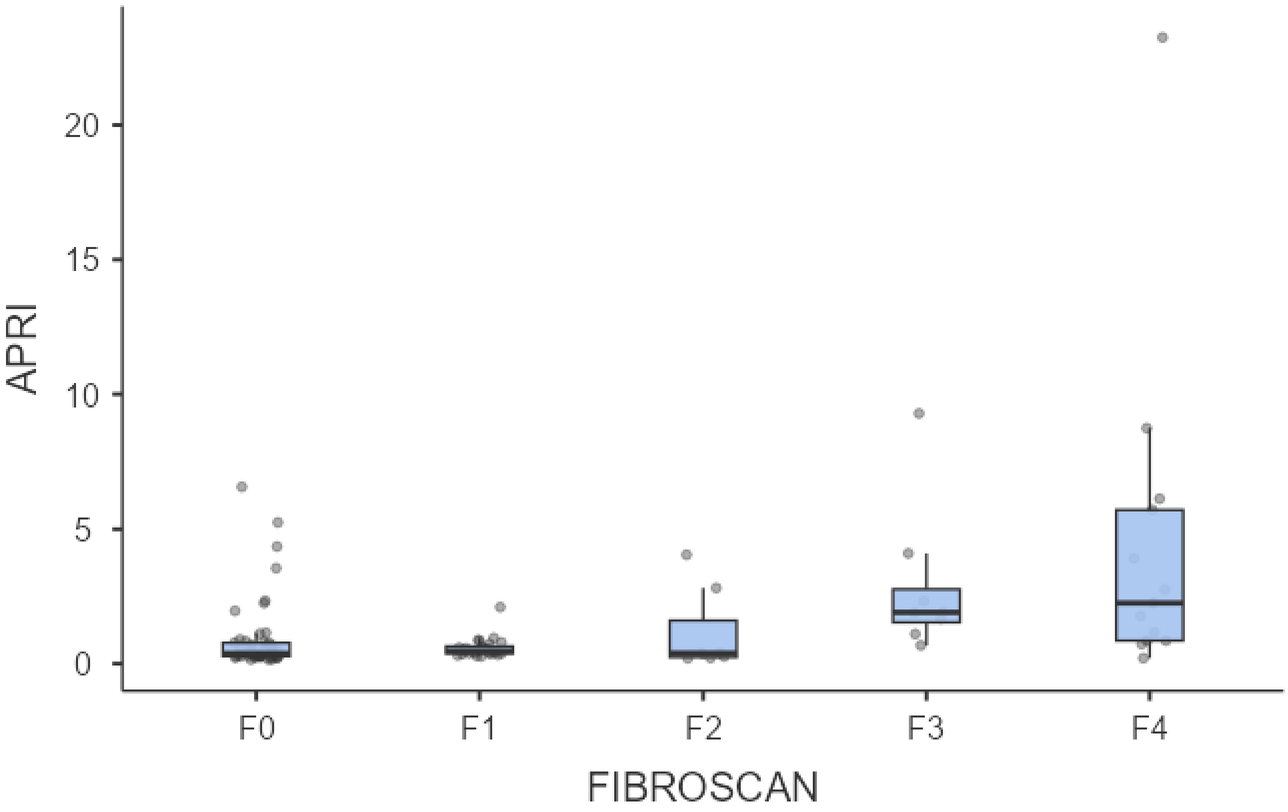

APRI
20
15
10
5
0
F0
F1
F2
F3
F4
FIBROSCAN

GPR

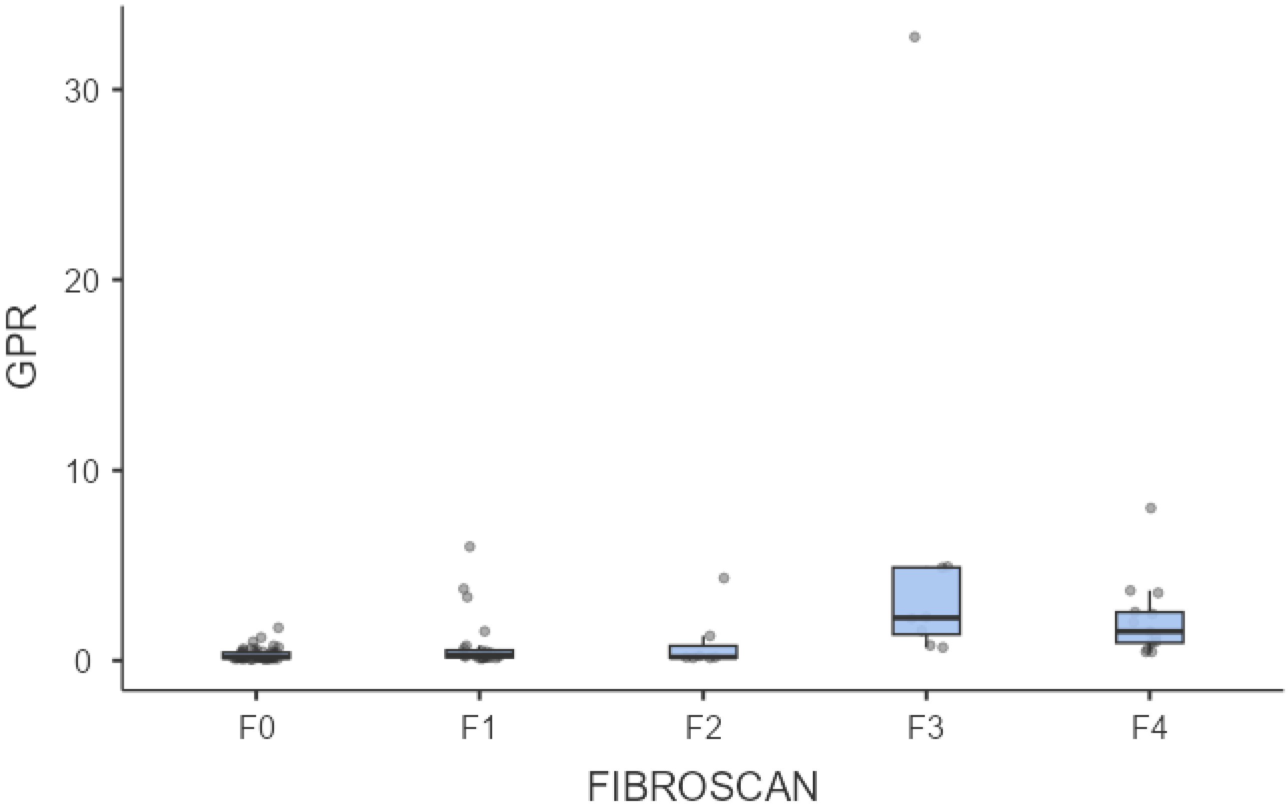

GPR
30
20
10
0
F0
F1
F2
F3
F4
FIBROSCAN

IgG

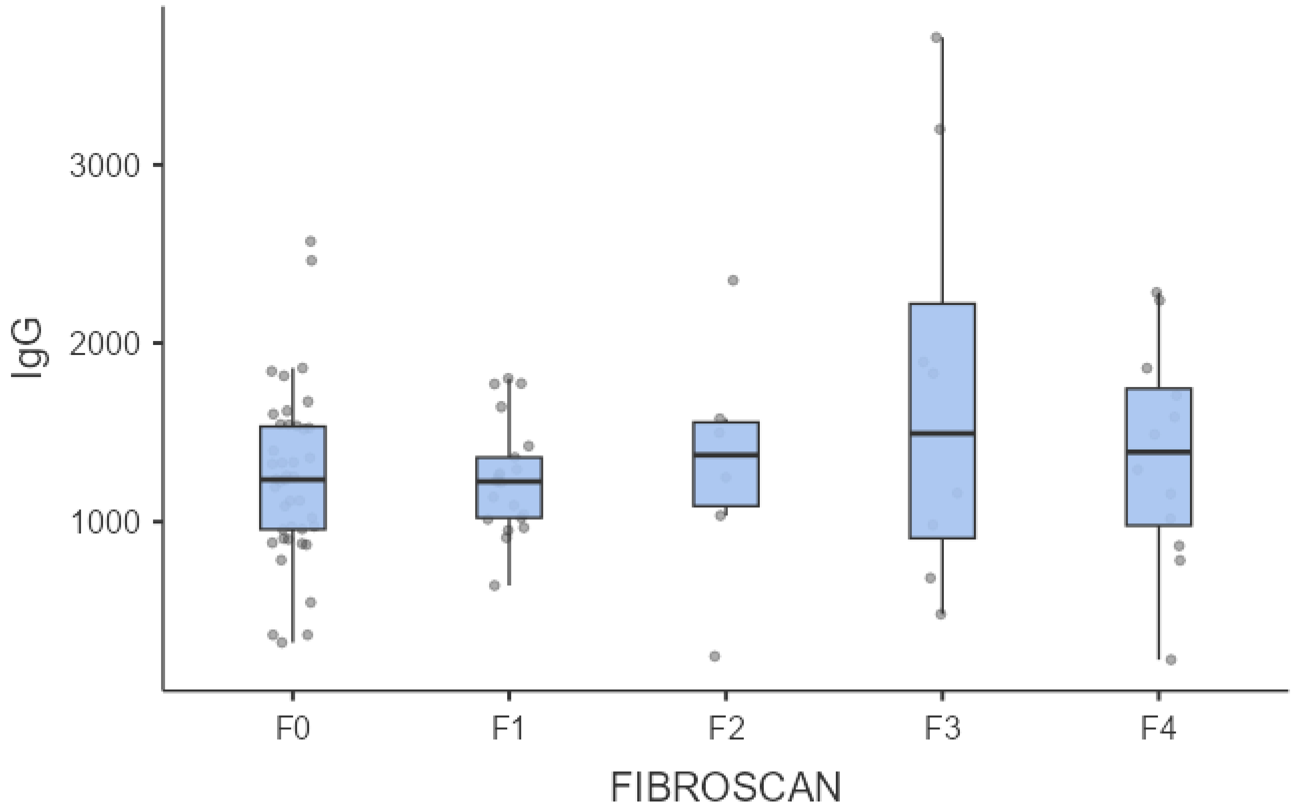

Albumin

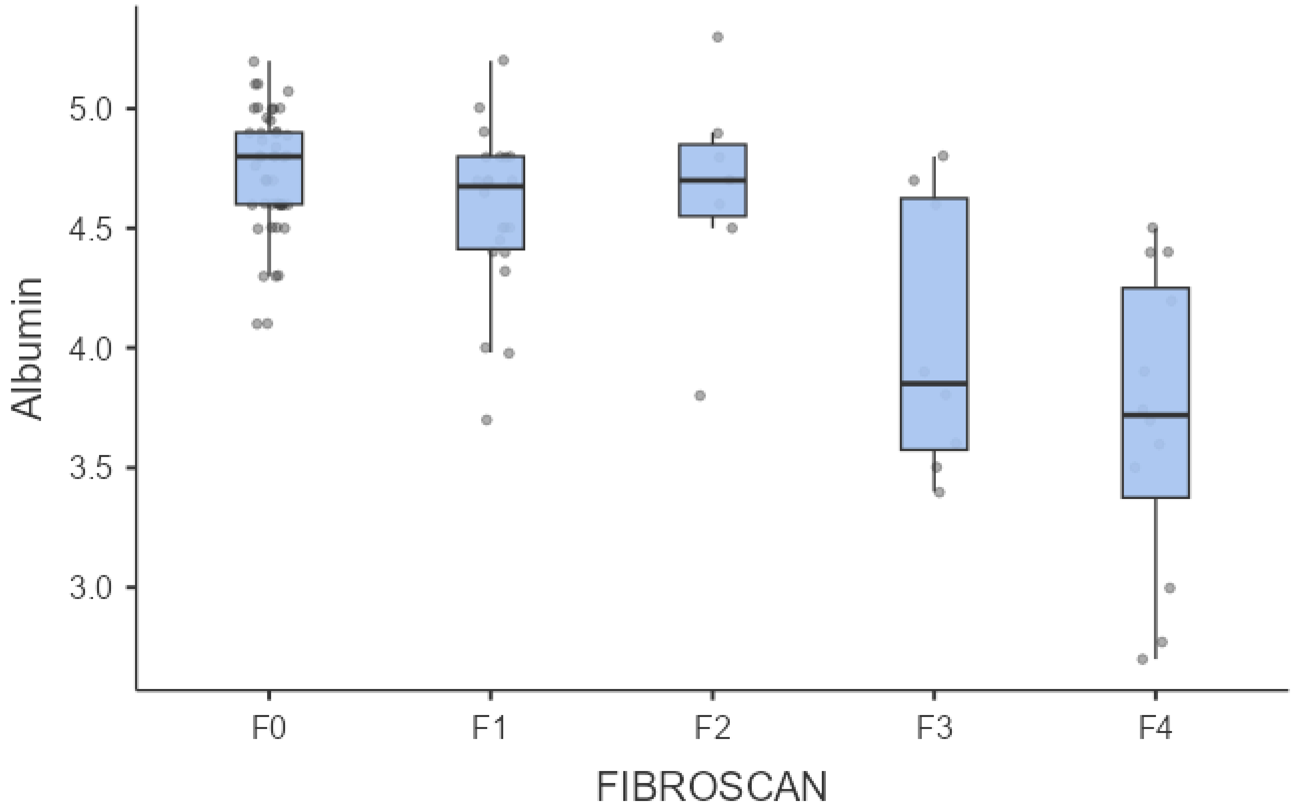

FibroIndex

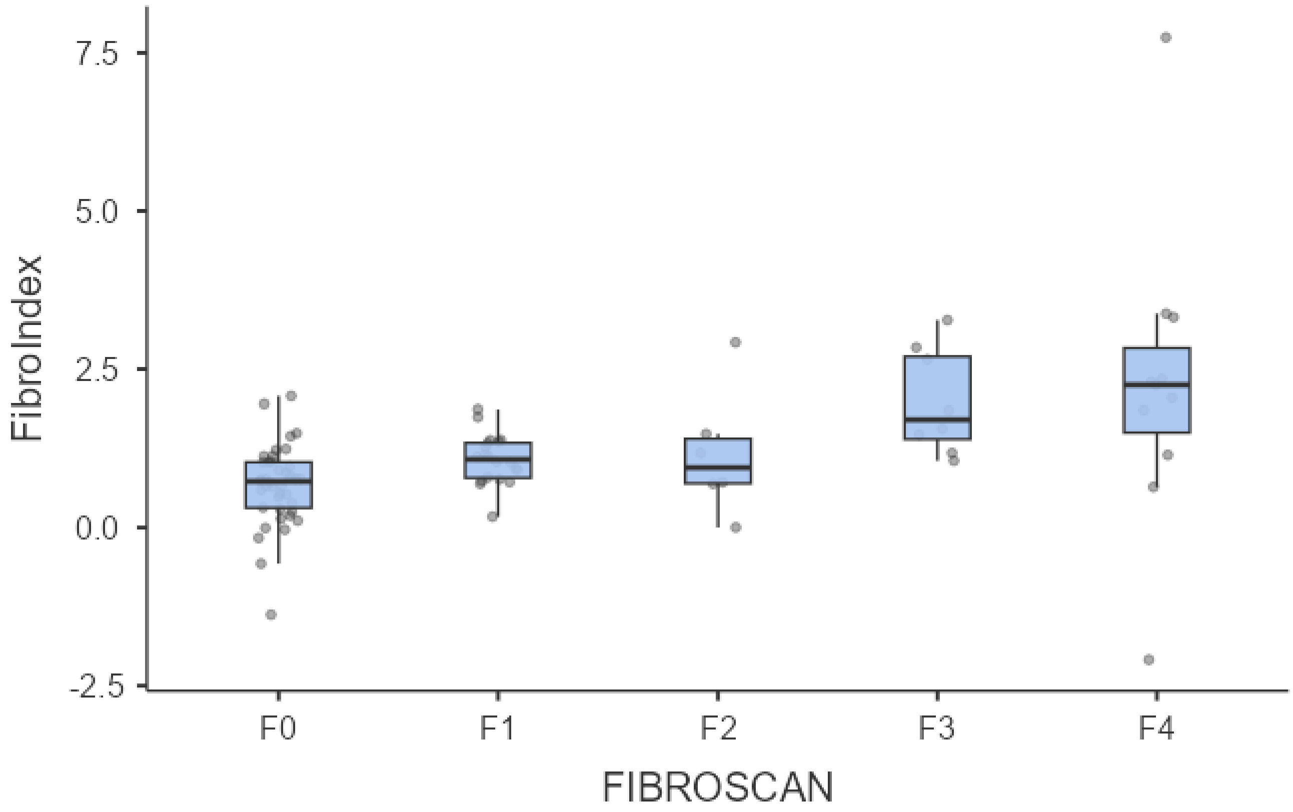

Boxplots with medians and IQR

horizontal bars indicate significant differences across the two groups they span

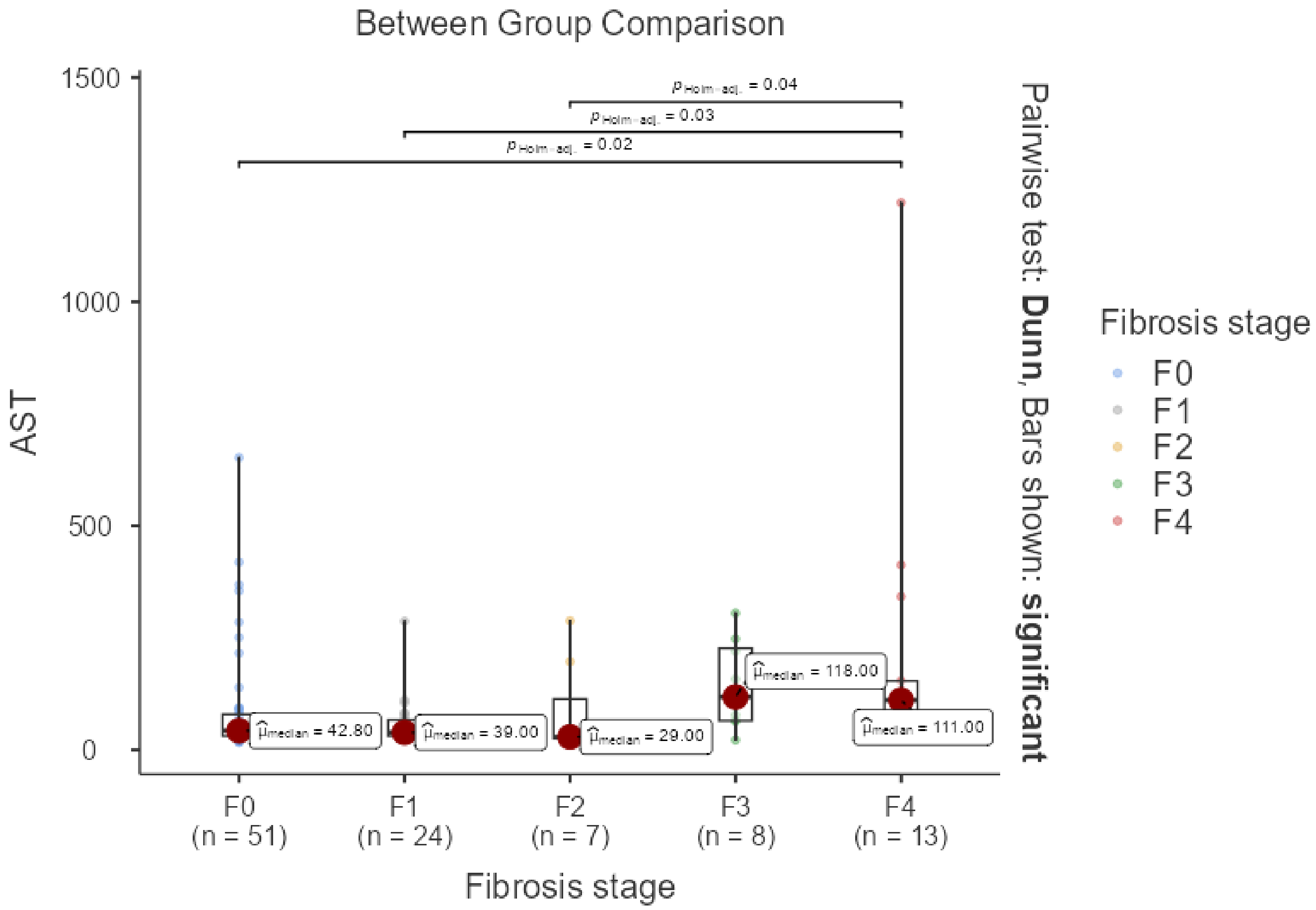

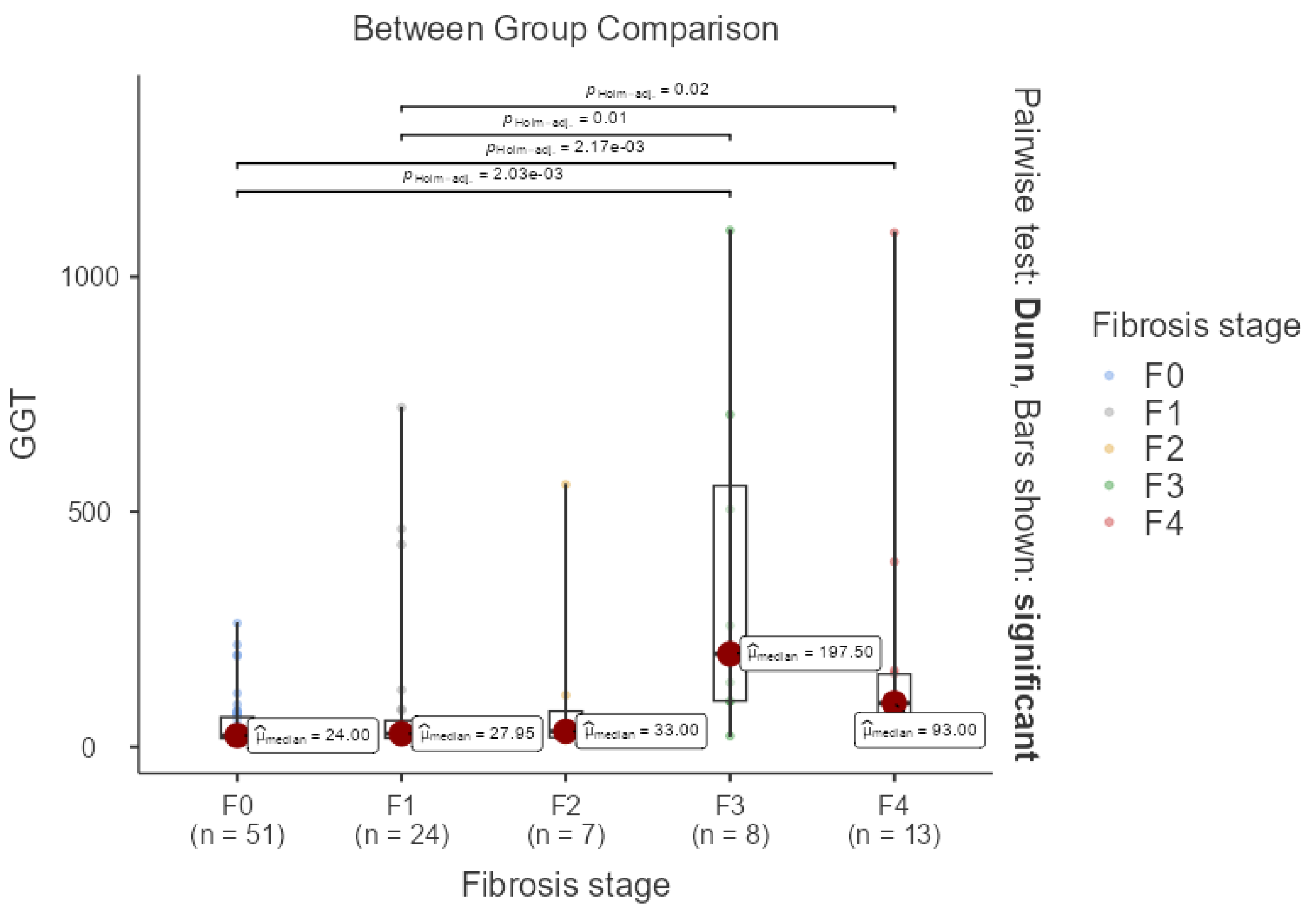

Between Group Comparison
$p_{Holm-adj.}$ = 0.02
$p_{Holm-adj.}$ = 0.01
$p_{Holm-adj.}$ = 2.17e-03
$p_{Holm-adj.}$ = 2.03e-03
GGT
1000
500
0
$\hat{\mu}_{median}$ = 24.00
$\hat{\mu}_{median}$ = 27.95
$\hat{\mu}_{median}$ = 33.00
$\hat{\mu}_{median}$ = 197.50
$\hat{\mu}_{median}$ = 93.00
F0 (n = 51)
F1 (n = 24)
F2 (n = 7)
F3 (n = 8)
F4 (n = 13)
Fibrosis stage
Pairwise test: **Dunn**, Bars shown: **significant**
Fibrosis stage
F0
F1
F2
F3
F4

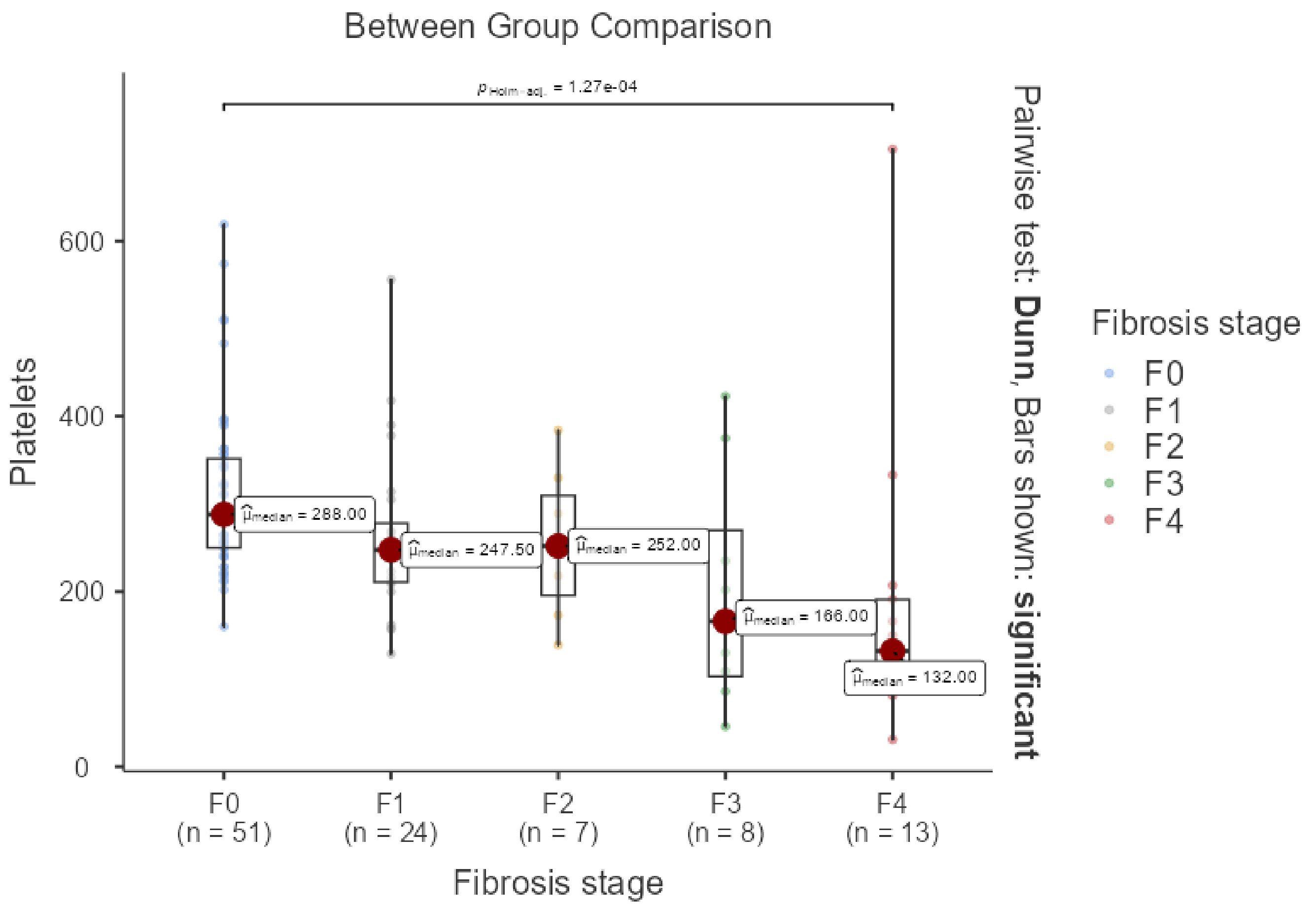

Between Group Comparison
$p_{Holm-adj.}$ = 1.27e-04
Platelets
600
400
200
0
$\hat{\mu}_{median}$ = 288.00
$\hat{\mu}_{median}$ = 247.50
$\hat{\mu}_{median}$ = 252.00
$\hat{\mu}_{median}$ = 166.00
$\hat{\mu}_{median}$ = 132.00
F0 (n = 51)
F1 (n = 24)
F2 (n = 7)
F3 (n = 8)
F4 (n = 13)
Fibrosis stage
Pairwise test: **Dunn**, Bars shown: **significant**
Fibrosis stage
F0
F1
F2
F3
F4

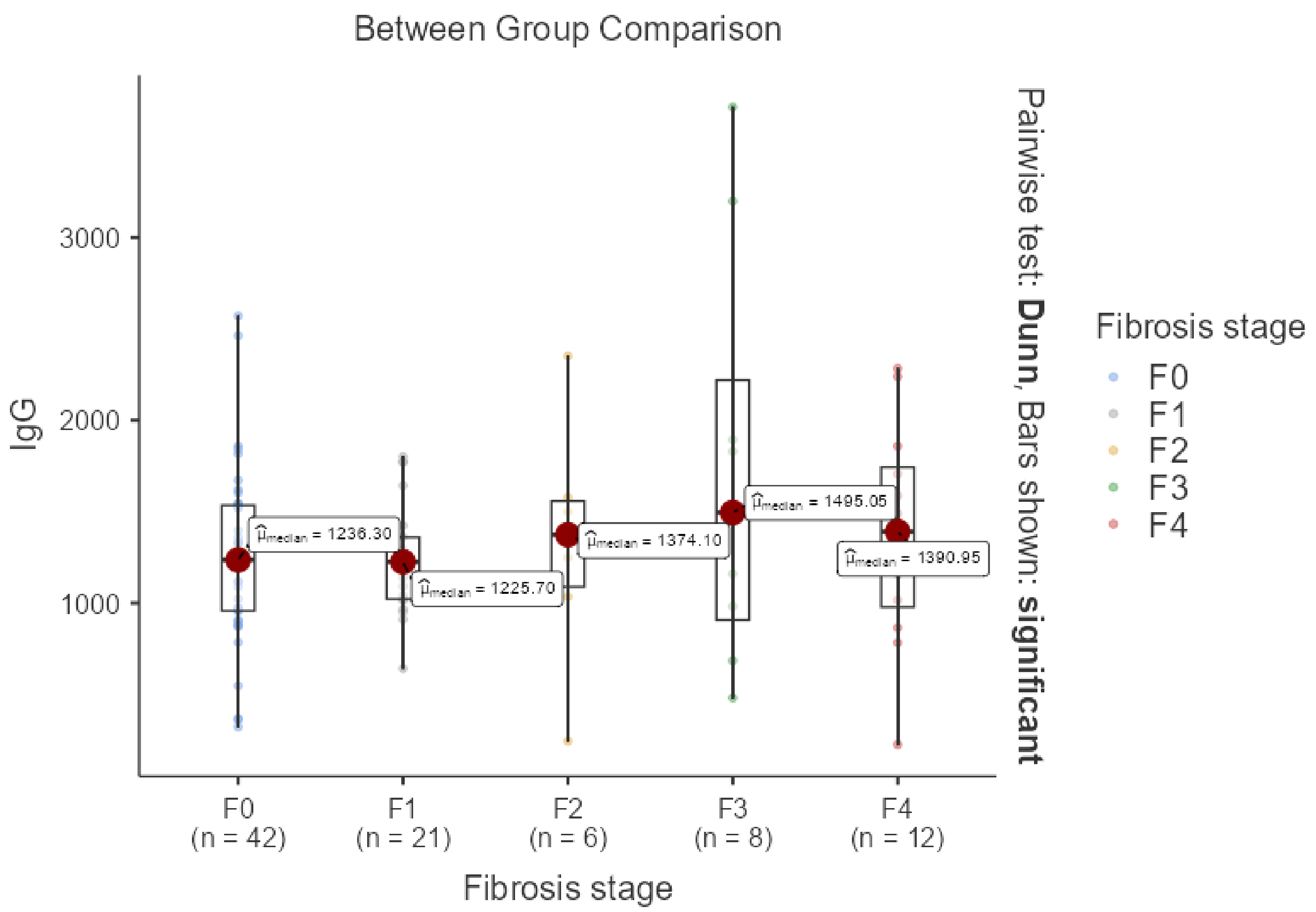
Between Group Comparison
IgG
3000
2000
1000
$\widehat{\mu}_{median}$ = 1236.30
$\widehat{\mu}_{median}$ = 1225.70
$\widehat{\mu}_{median}$ = 1374.10
$\widehat{\mu}_{median}$ = 1495.05
$\widehat{\mu}_{median}$ = 1390.95
F0 (n = 42)
F1 (n = 21)
F2 (n = 6)
F3 (n = 8)
F4 (n = 12)
Fibrosis stage
Pairwise test: **Dunn**, Bars shown: **significant**
Fibrosis stage
F0
F1
F2
F3
F4

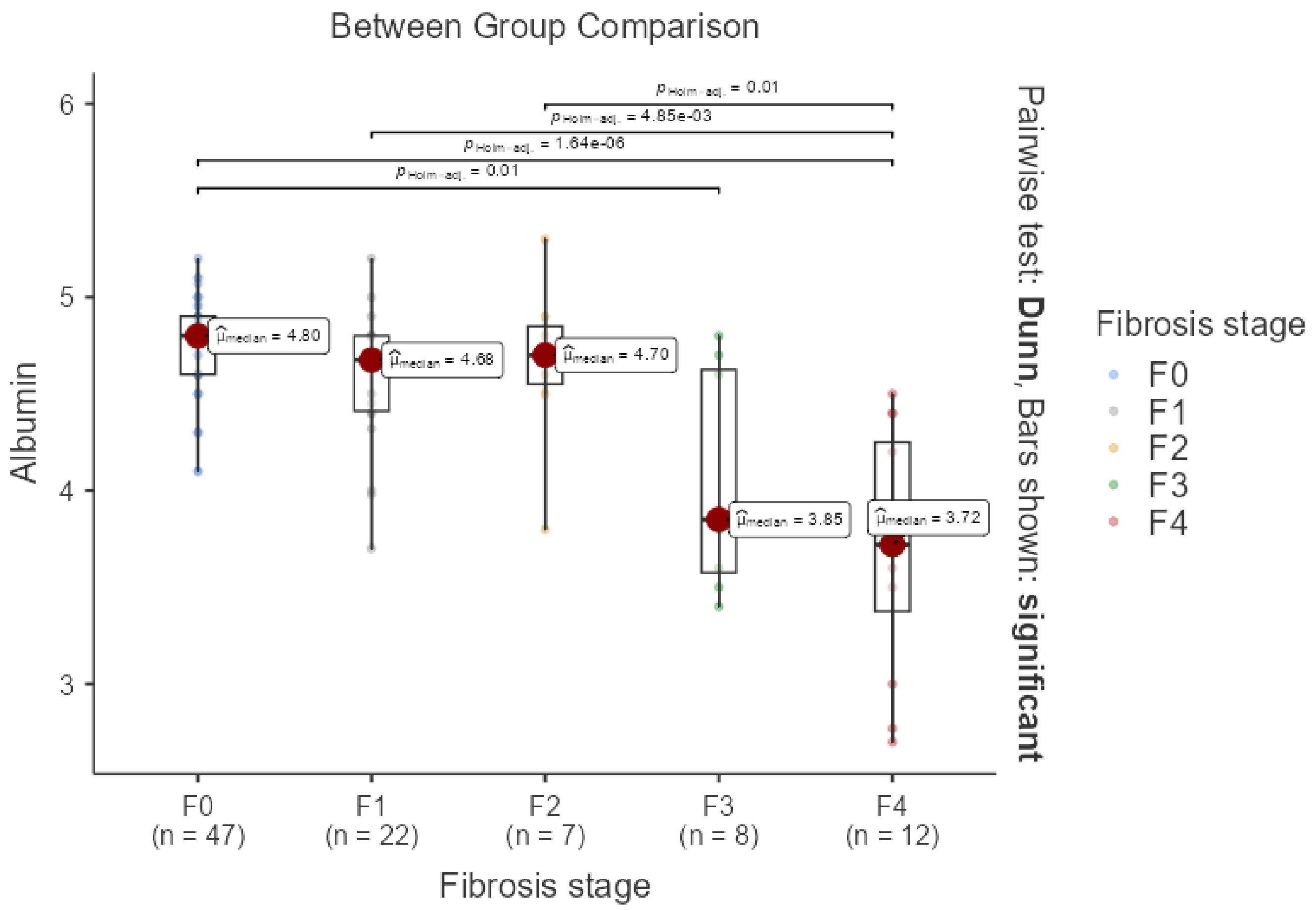
Between Group Comparison
Albumin
6
5
4
3
$p_{Holm-adj.}$ = 0.01
$p_{Holm-adj.}$ = 4.85e-03
$p_{Holm-adj.}$ = 1.64e-06
$p_{Holm-adj.}$ = 0.01
$\widehat{\mu}_{median}$ = 4.80
$\widehat{\mu}_{median}$ = 4.68
$\widehat{\mu}_{median}$ = 4.70
$\widehat{\mu}_{median}$ = 3.85
$\widehat{\mu}_{median}$ = 3.72
F0 (n = 47)
F1 (n = 22)
F2 (n = 7)
F3 (n = 8)
F4 (n = 12)
Fibrosis stage
Pairwise test: **Dunn**, Bars shown: **significant**
Fibrosis stage
F0
F1
F2
F3
F4

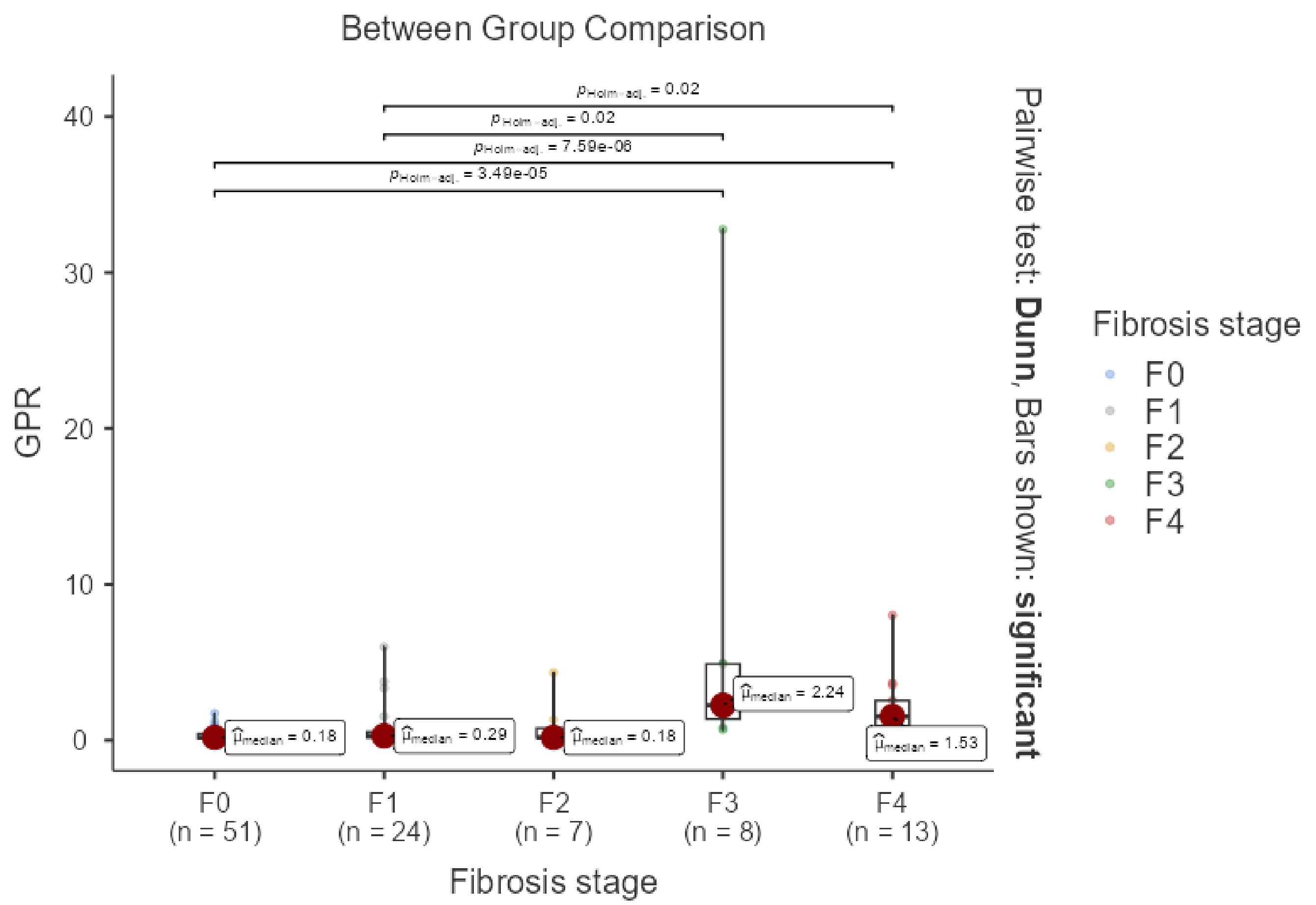

Between Group Comparison
$p_{Holm-adj.}$ = 0.02
$p_{Holm-adj.}$ = 0.02
$p_{Holm-adj.}$ = 7.59e-06
$p_{Holm-adj.}$ = 3.49e-05
GPR
$\hat{\mu}_{median}$ = 0.18
$\hat{\mu}_{median}$ = 0.29
$\hat{\mu}_{median}$ = 0.18
$\hat{\mu}_{median}$ = 2.24
$\hat{\mu}_{median}$ = 1.53
F0 (n = 51)
F1 (n = 24)
F2 (n = 7)
F3 (n = 8)
F4 (n = 13)
Fibrosis stage
Pairwise test: **Dunn**, Bars shown: **significant**
Fibrosis stage
F0
F1
F2
F3
F4

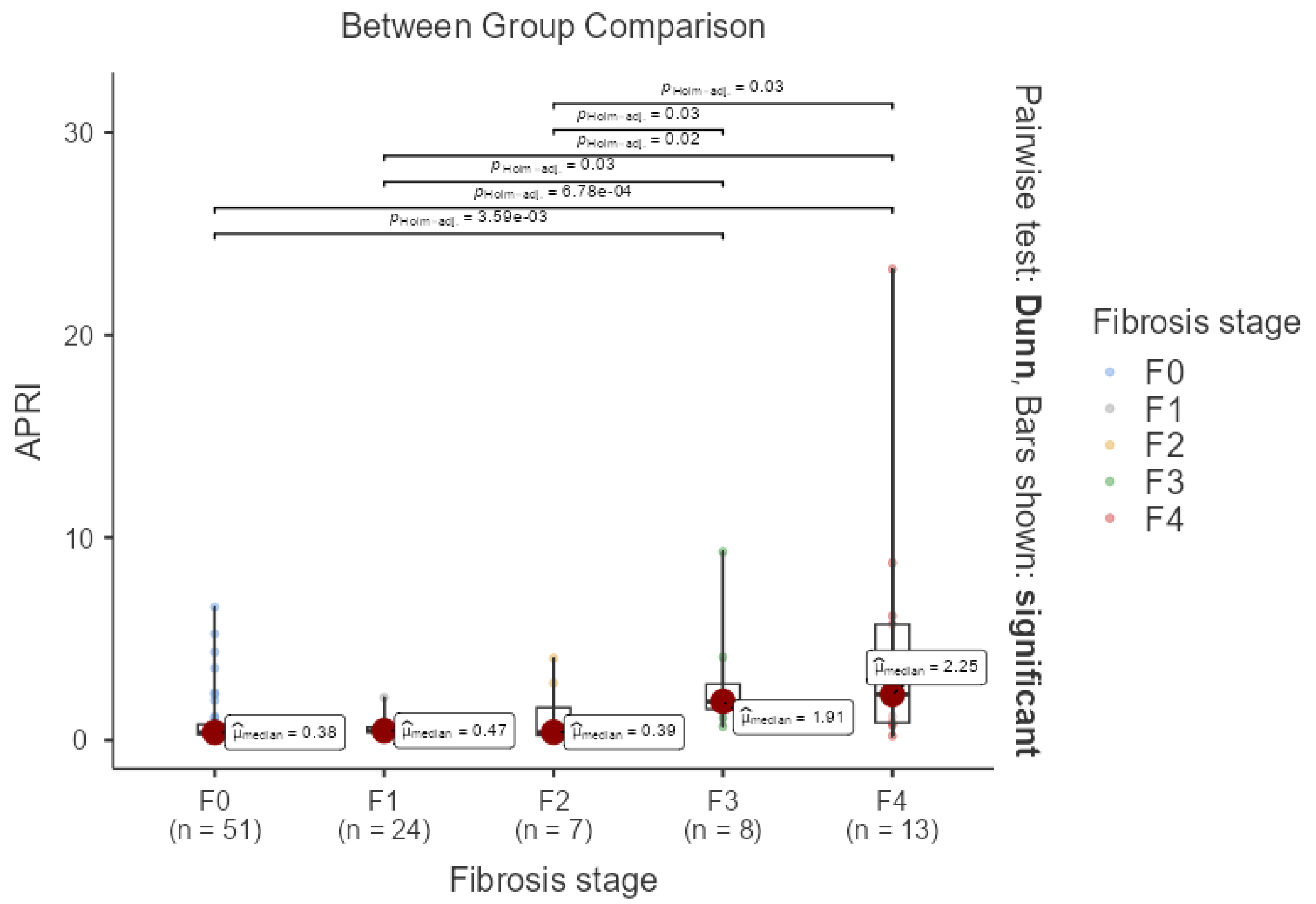

Between Group Comparison
$p_{Holm-adj.}$ = 0.03
$p_{Holm-adj.}$ = 0.03
$p_{Holm-adj.}$ = 0.02
$p_{Holm-adj.}$ = 0.03
$p_{Holm-adj.}$ = 6.78e-04
$p_{Holm-adj.}$ = 3.59e-03
APRI
$\hat{\mu}_{median}$ = 0.38
$\hat{\mu}_{median}$ = 0.47
$\hat{\mu}_{median}$ = 0.39
$\hat{\mu}_{median}$ = 1.91
$\hat{\mu}_{median}$ = 2.25
F0 (n = 51)
F1 (n = 24)
F2 (n = 7)
F3 (n = 8)
F4 (n = 13)
Fibrosis stage
Pairwise test: **Dunn**, Bars shown: **significant**
Fibrosis stage
F0
F1
F2
F3
F4

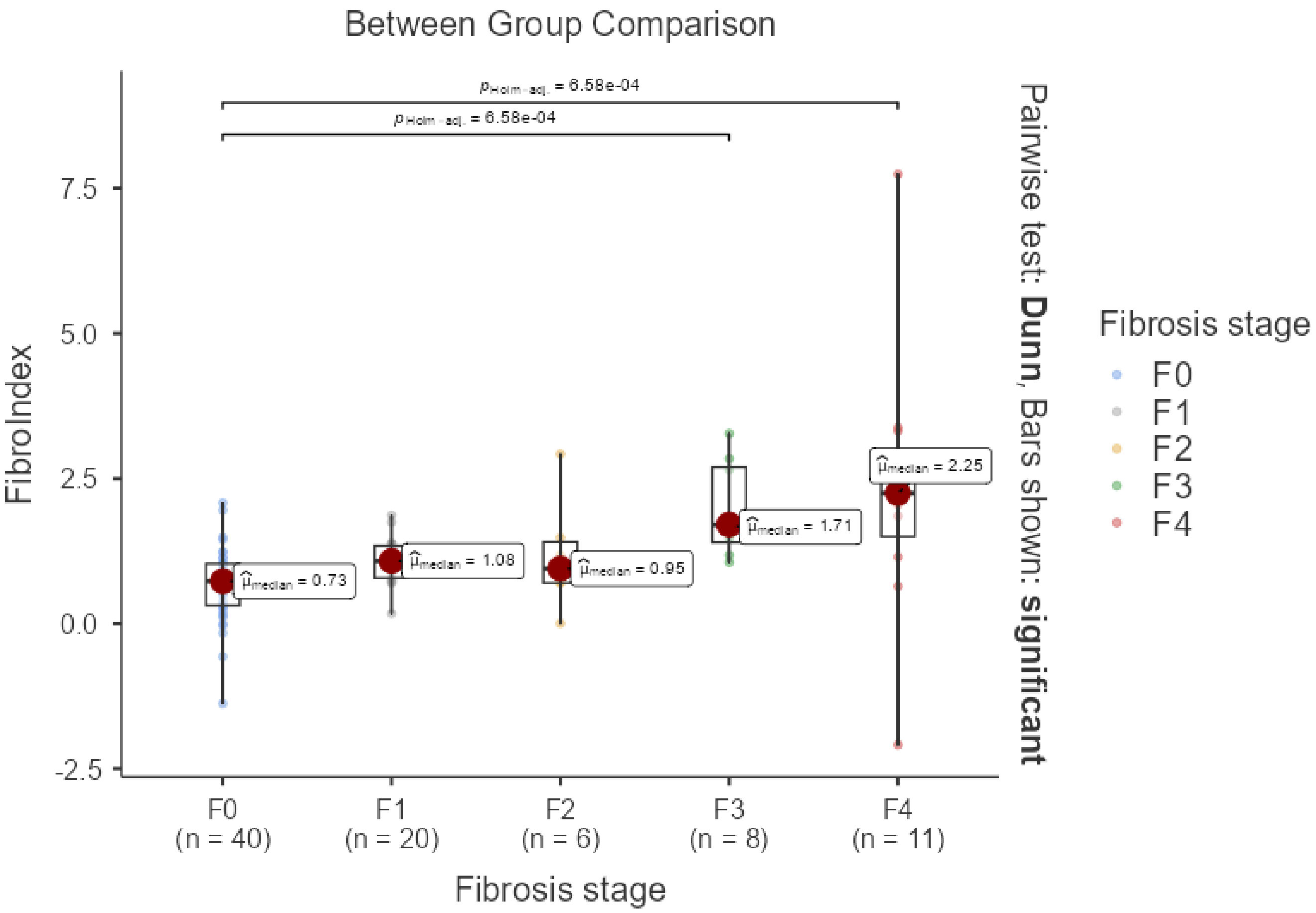

Between Group Comparison
$p_{Holm-adj.}$ = 6.58e-04
$p_{Holm-adj.}$ = 6.58e-04
FibroIndex
7.5
5.0
2.5
0.0
-2.5
$\hat{\mu}_{median}$ = 0.73
$\hat{\mu}_{median}$ = 1.08
$\hat{\mu}_{median}$ = 0.95
$\hat{\mu}_{median}$ = 1.71
$\hat{\mu}_{median}$ = 2.25
F0 (n = 40)
F1 (n = 20)
F2 (n = 6)
F3 (n = 8)
F4 (n = 11)
Fibrosis stage
Pairwise test: **Dunn**, Bars shown: **significant**
Fibrosis stage
F0
F1
F2
F3
F4
