# Supplementary material for: Performance of Blood-Based Indirect Scores Compared to Transient Elastography in Children with Chronic Liver Disease
Source: Diagnostics (Basel). 2026 Apr 6;16(7):1102. doi: 10.3390/diagnostics16071102 (PMC13074151; doi:10.3390/diagnostics16071102)

Supplementary figure S4 – Bar charts of etiologies and fibrosis staging across age groups

Stacked bar chart (proportions) of etiologies and fibrosis staging across age groups

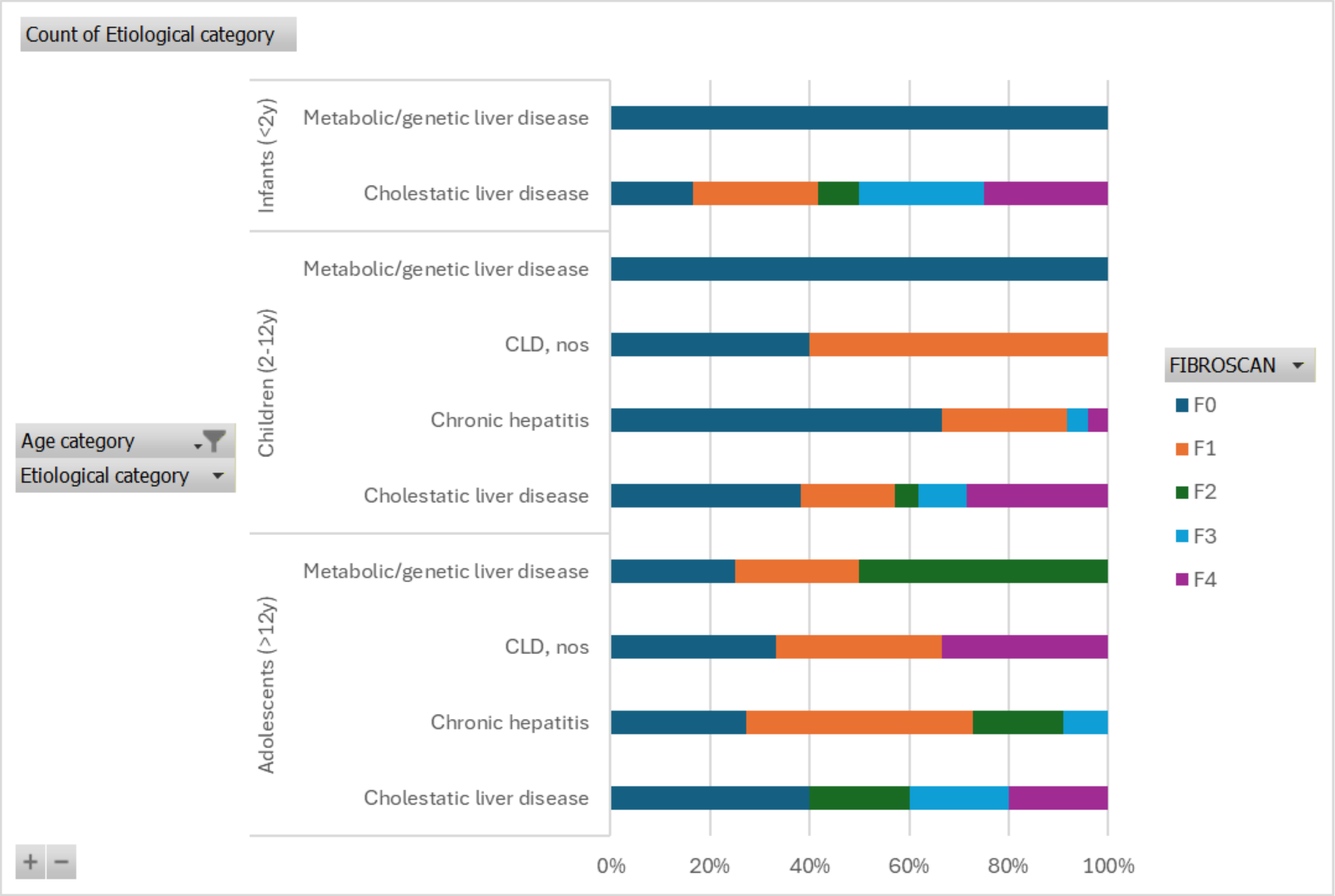

Bar chart (absolute counts) of etiologies and fibrosis staging across age groups

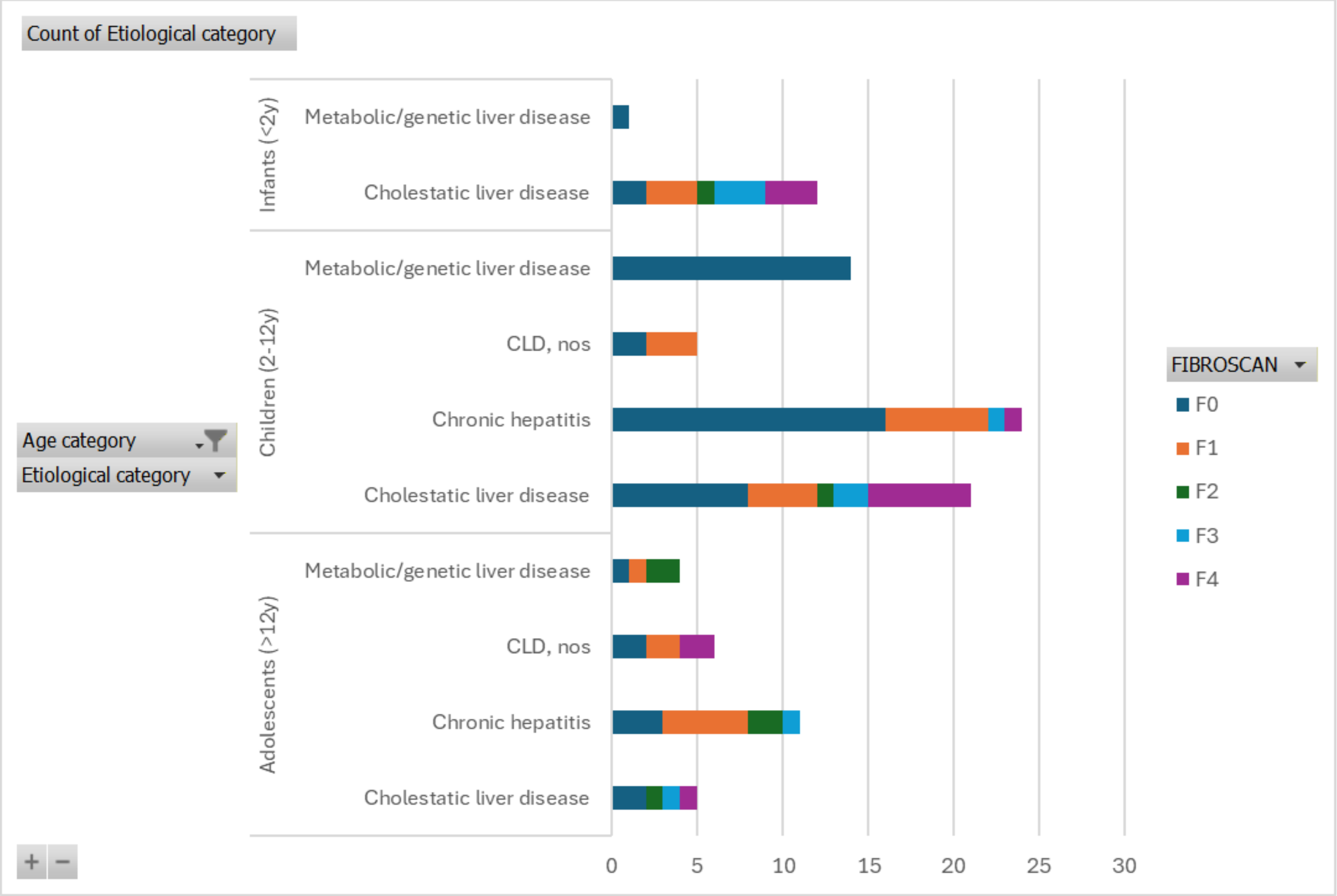

Supplement: Supplementary file 1 [file diagnostics-16-01102-s001.zip › Supplementary Figure S4 bar charts of etiologies and fibrosis staging across age groups.pdf]
