# Supplementary material for: Performance of Blood-Based Indirect Scores Compared to Transient Elastography in Children with Chronic Liver Disease
Source: Diagnostics (Basel). 2026 Apr 6;16(7):1102. doi: 10.3390/diagnostics16071102 (PMC13074151; doi:10.3390/diagnostics16071102)

Supplementary Figures S1a-q for parameters compared between groups (F0-2 versus F3-4 groups)

Supplementary Figure S1a- Age (months)

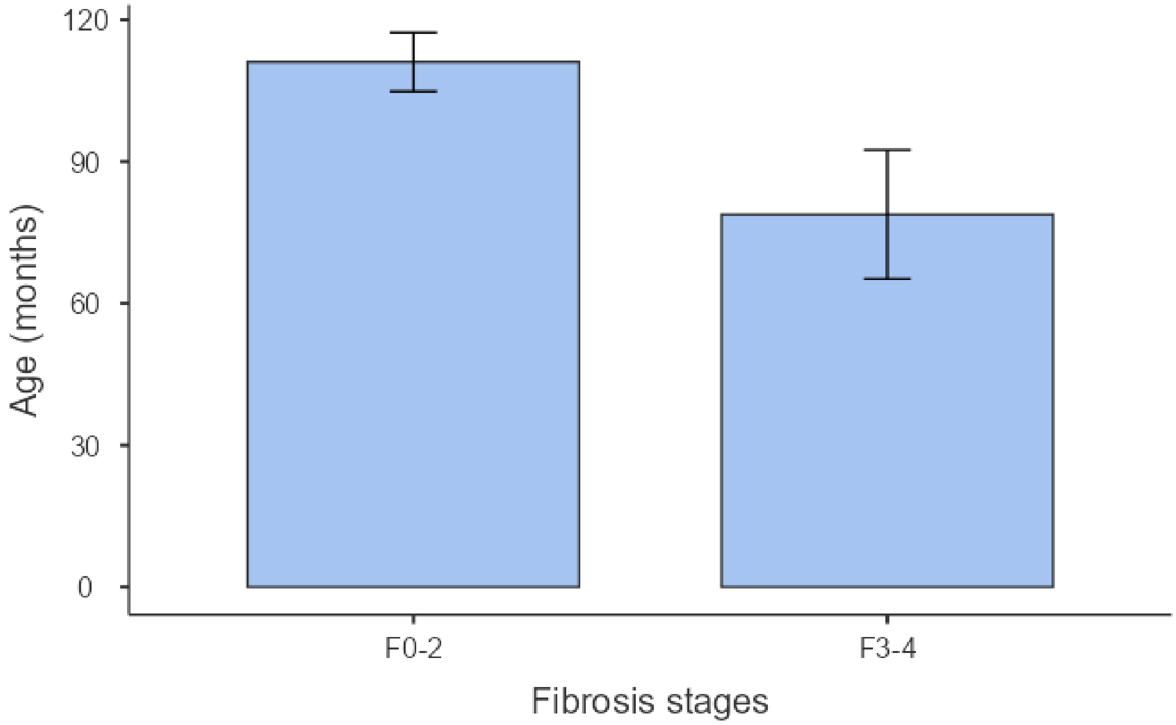

Supplementary Figure S1b- Weight (kg)

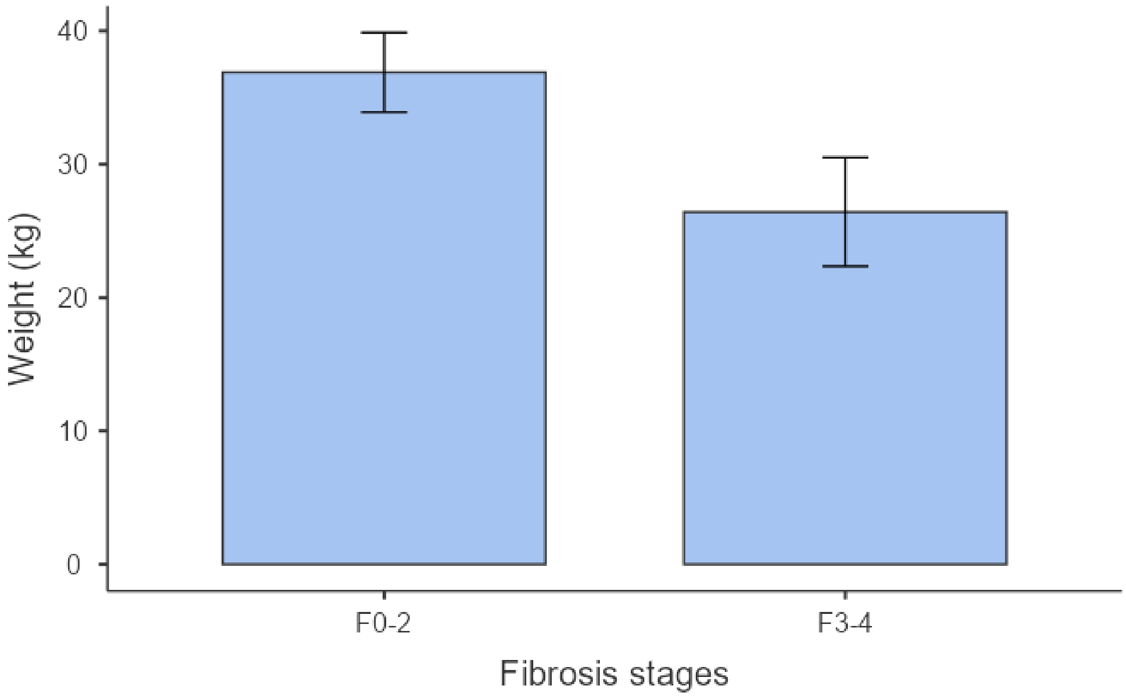

Supplementary Figure S1c- Height (cm)

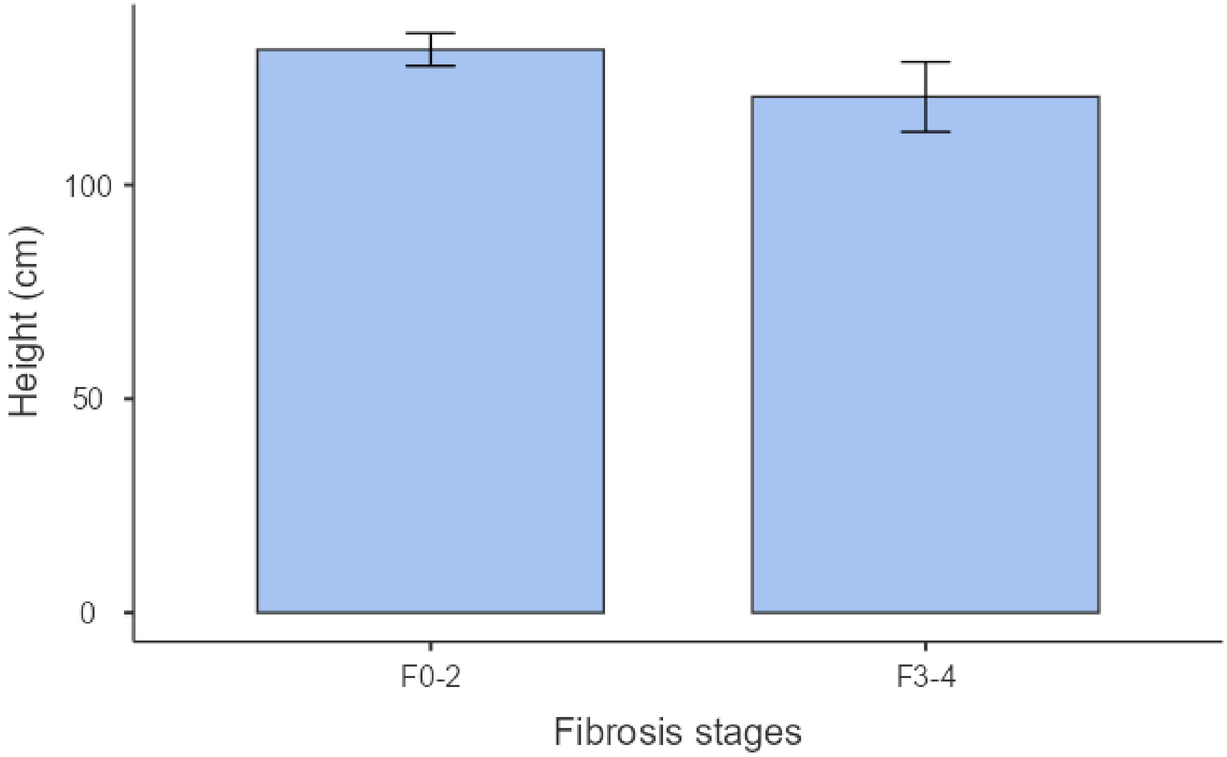

Supplementary Figure S1d – White blood cells (WBC , 10^9/liter)

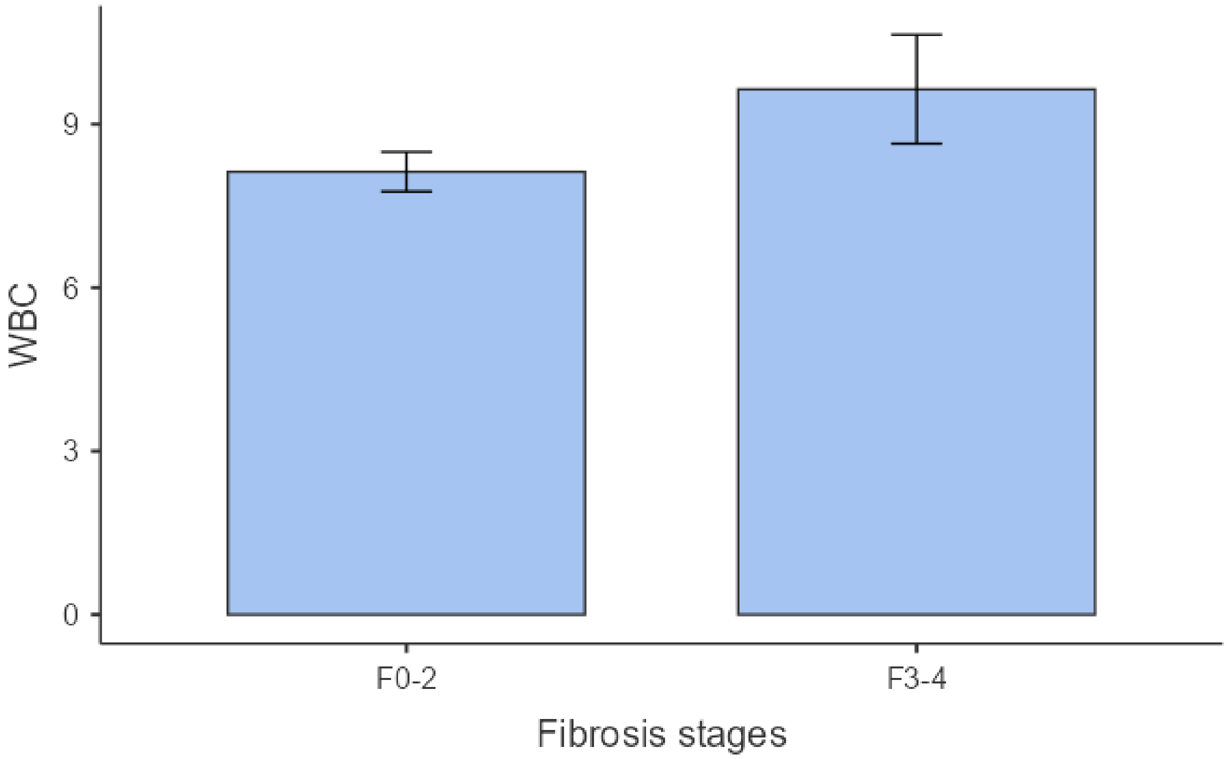

Supplementary Figure S1e – Red blod cells (RBC, 10^{12}/liter)

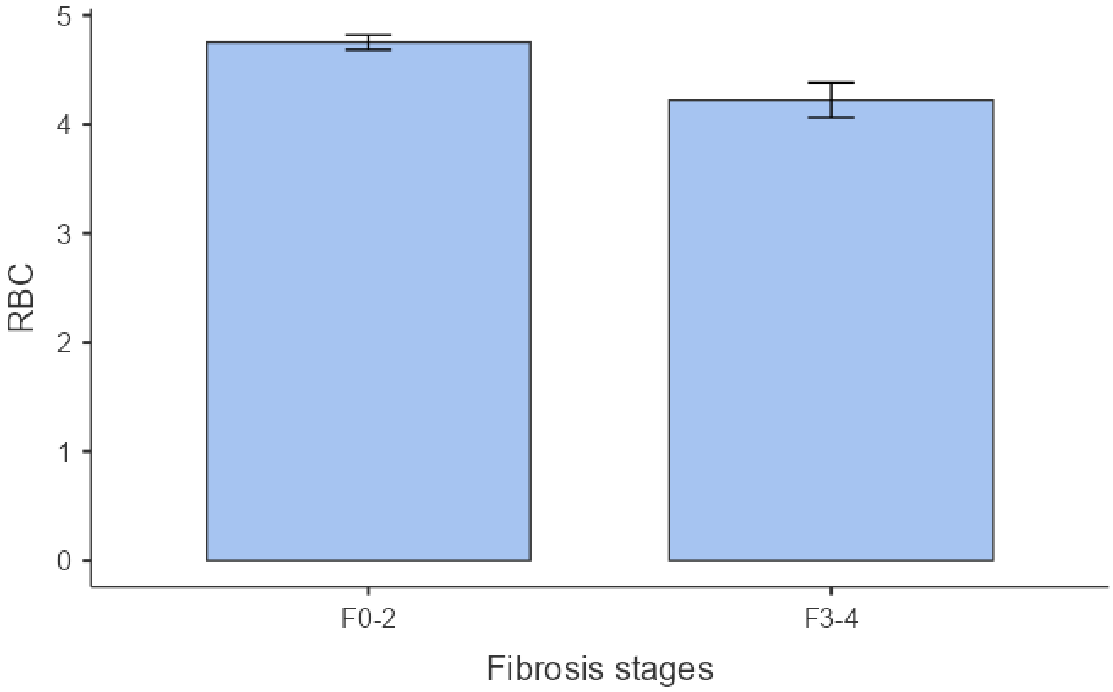

Supplementary Figure S1f - Hemoglobin (g/dL)

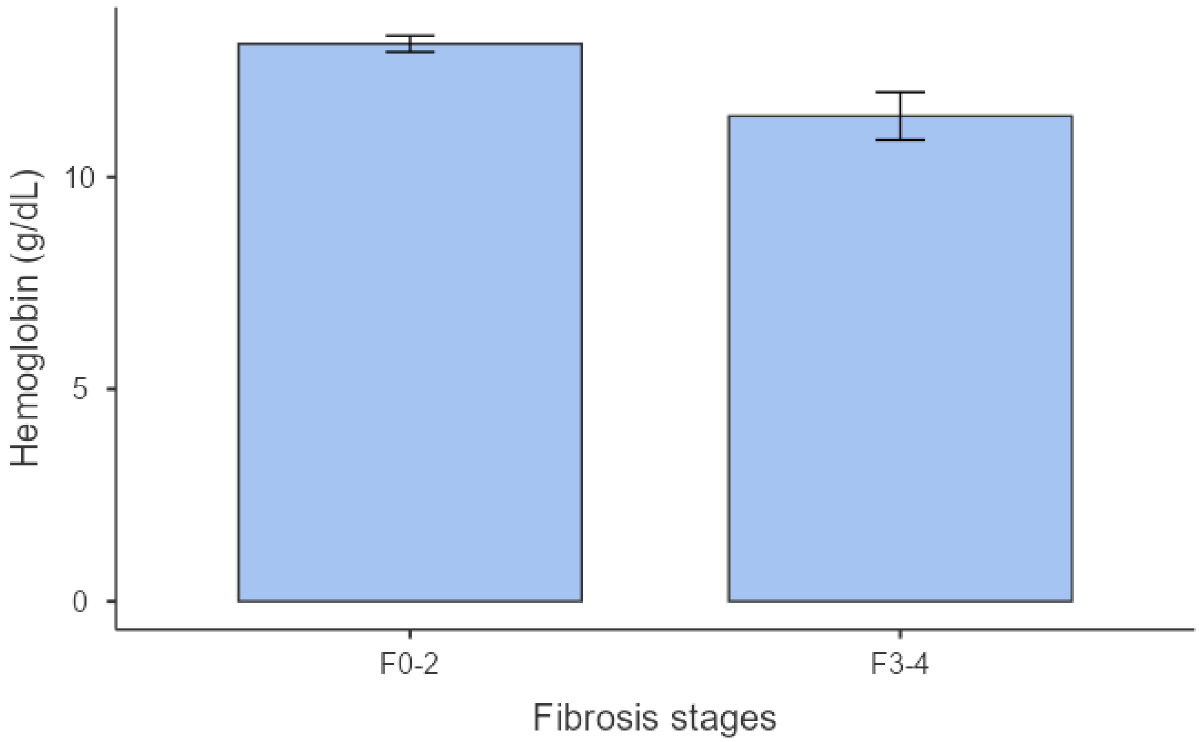

Supplementary Figure S1g - Platelets (10^9/liter)

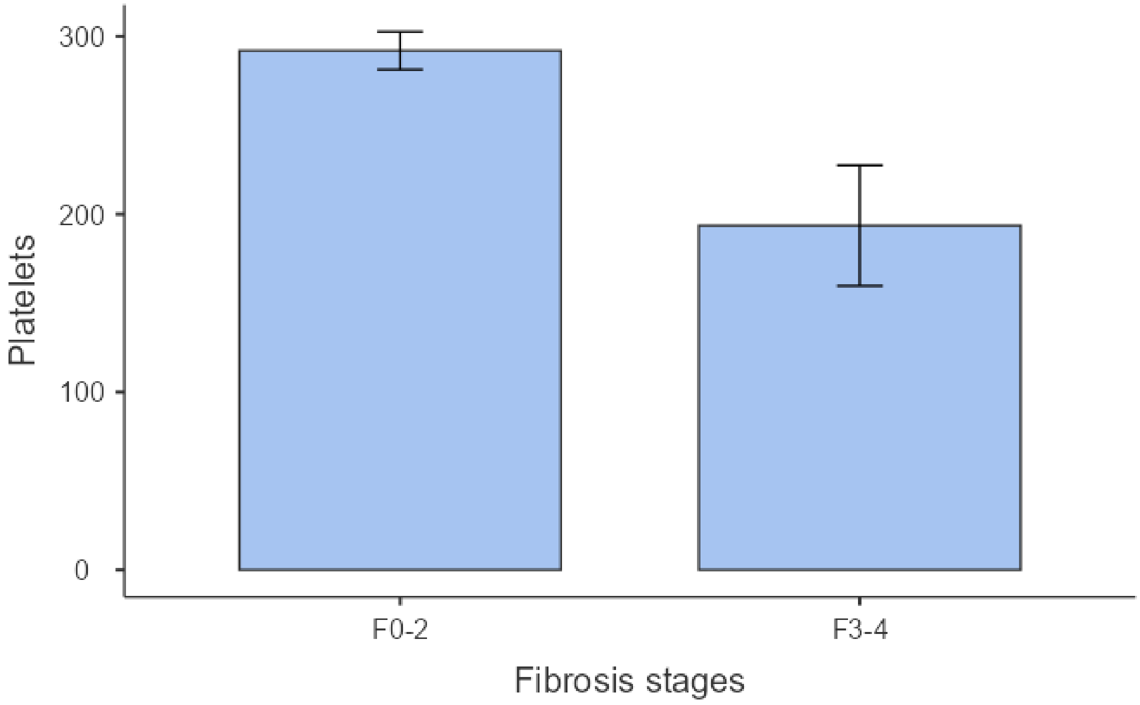

Supplementary Figure S1h – International Normalized Ratio (INR)

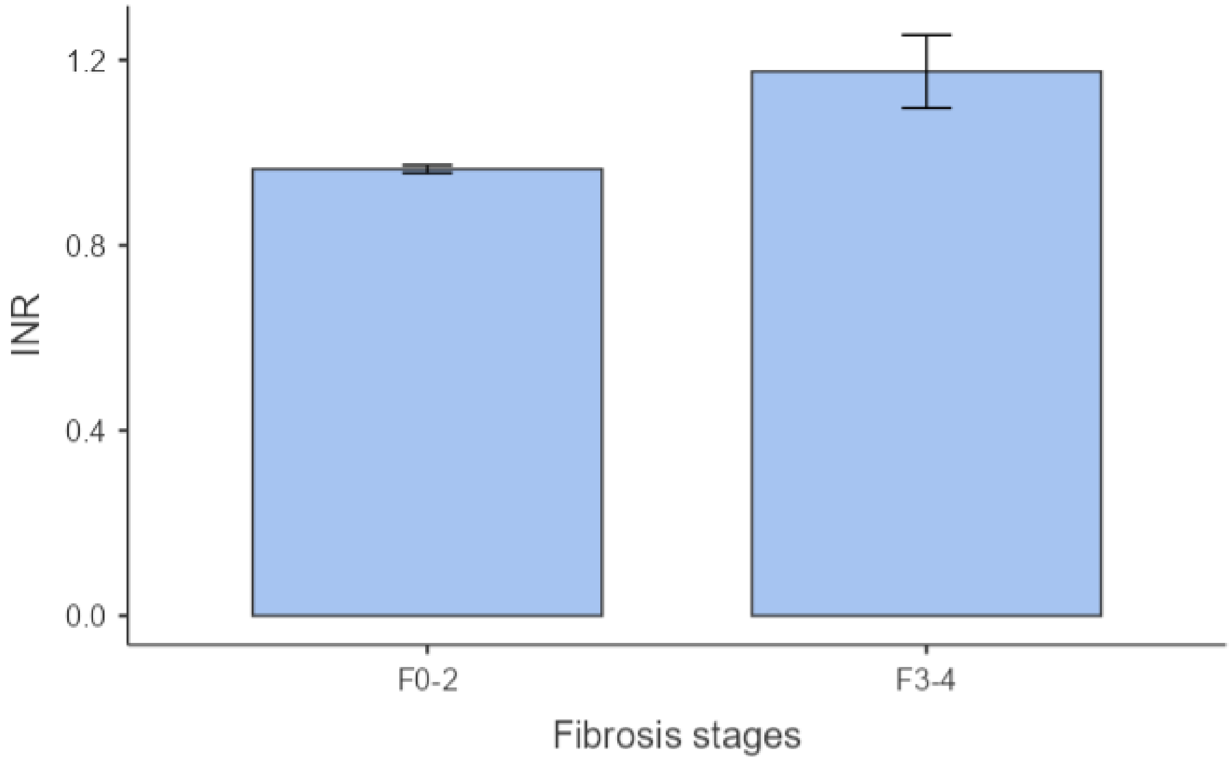

Supplementary Figure S1i – Aspartate aminotransferase (AST , U/L)

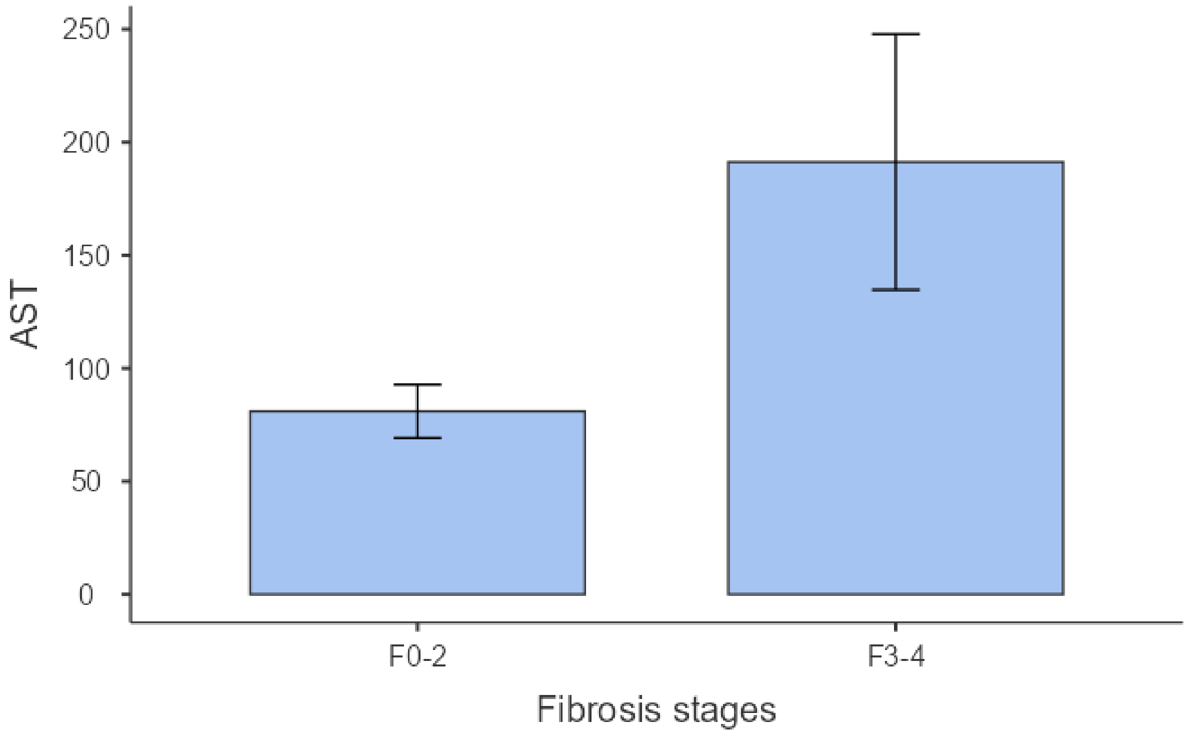

Supplementary Figure S1j – Alanine aminotransferase (ALT, U/L)

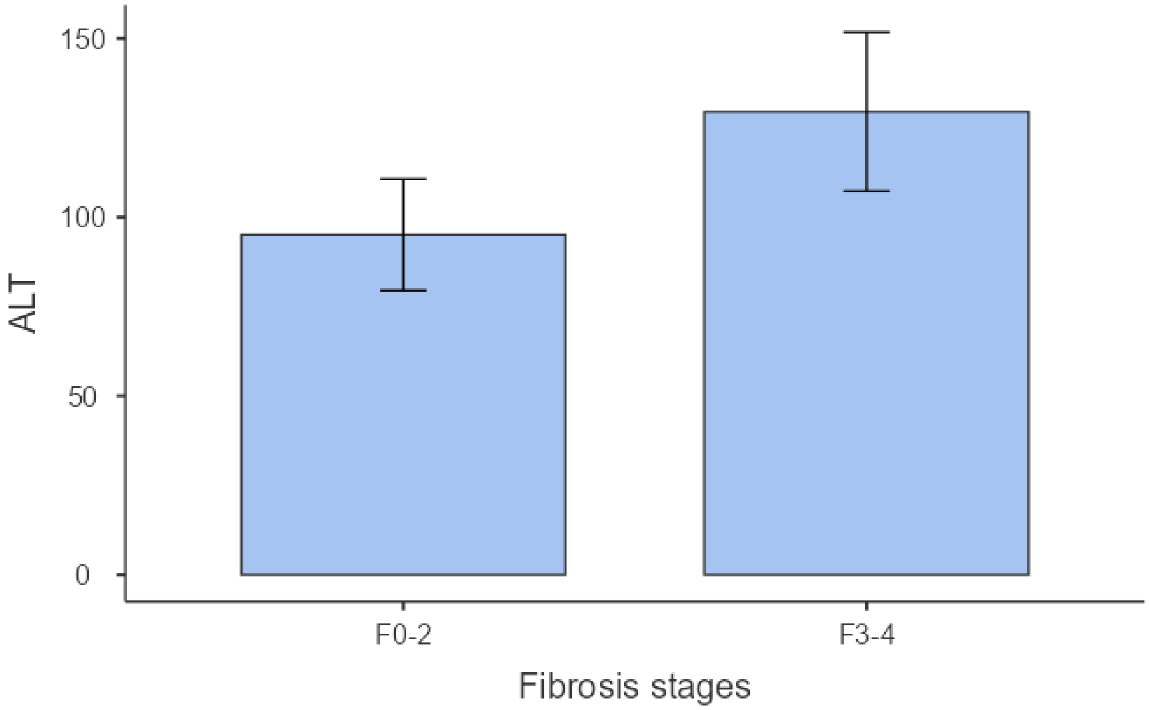

Supplementary Figure S1k – Gamma Glutamyl transferase (GGT, U/L)

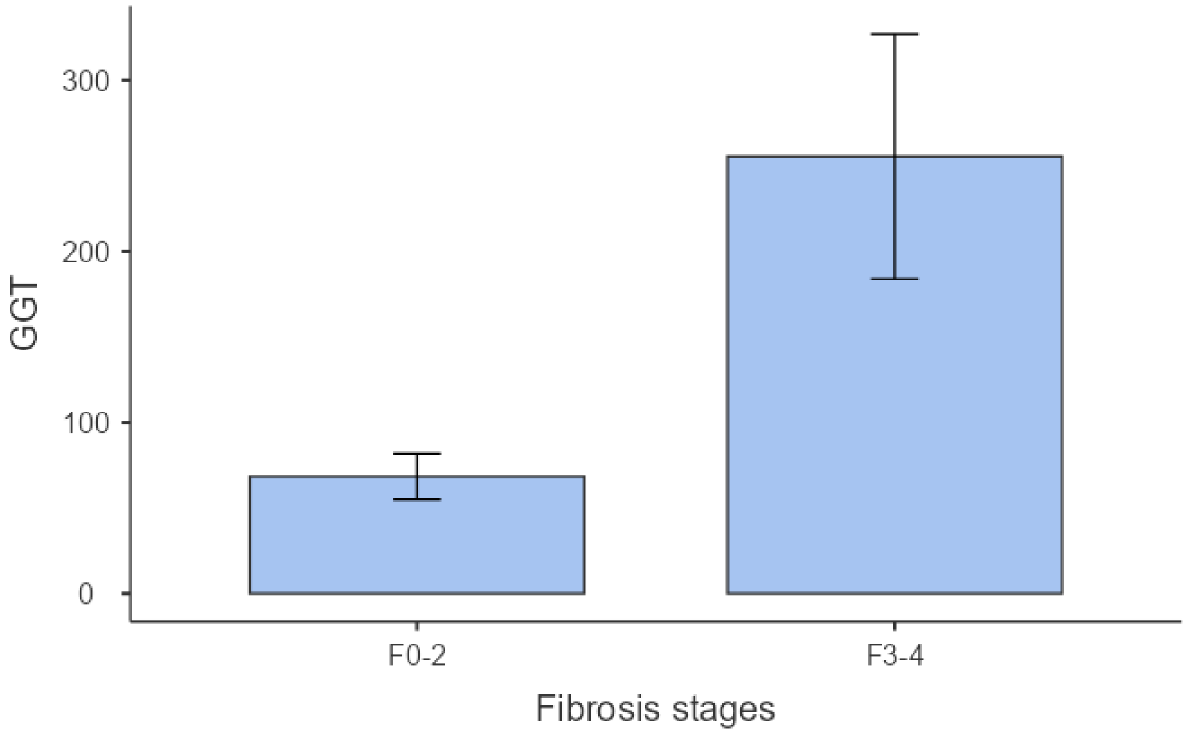

Supplementary Figure S1l - Total Bilirubin (mg/dL)

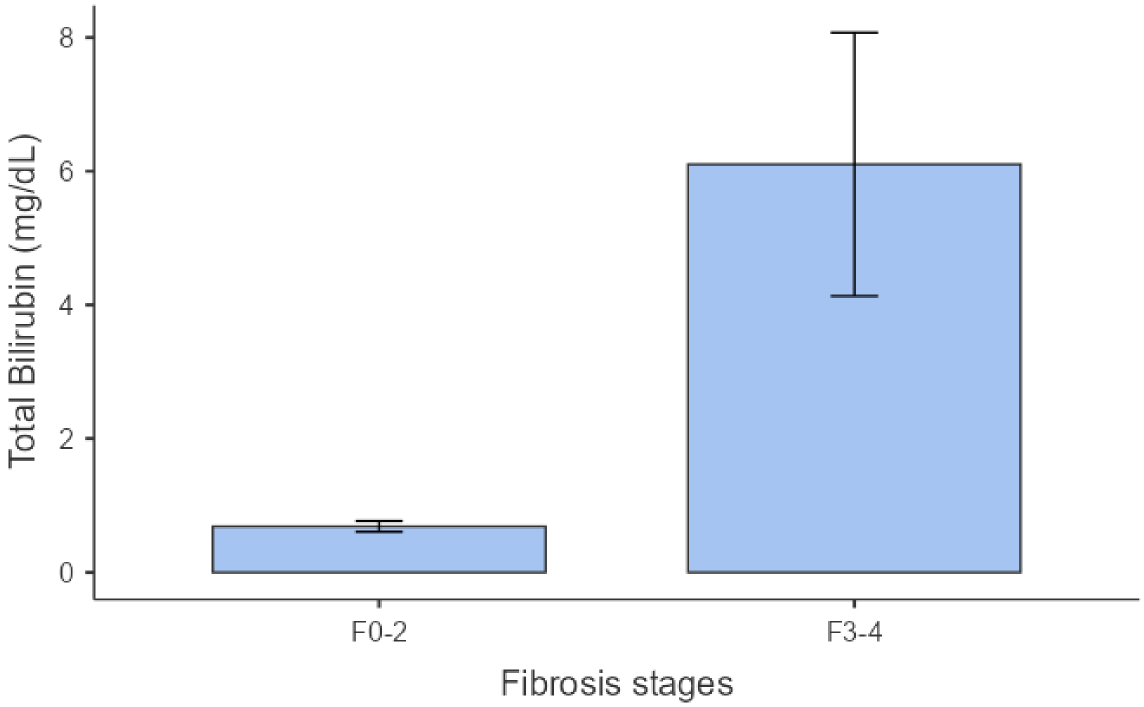

Supplementary Figure S1m - Conjugated Bilirubin (mg/dL)

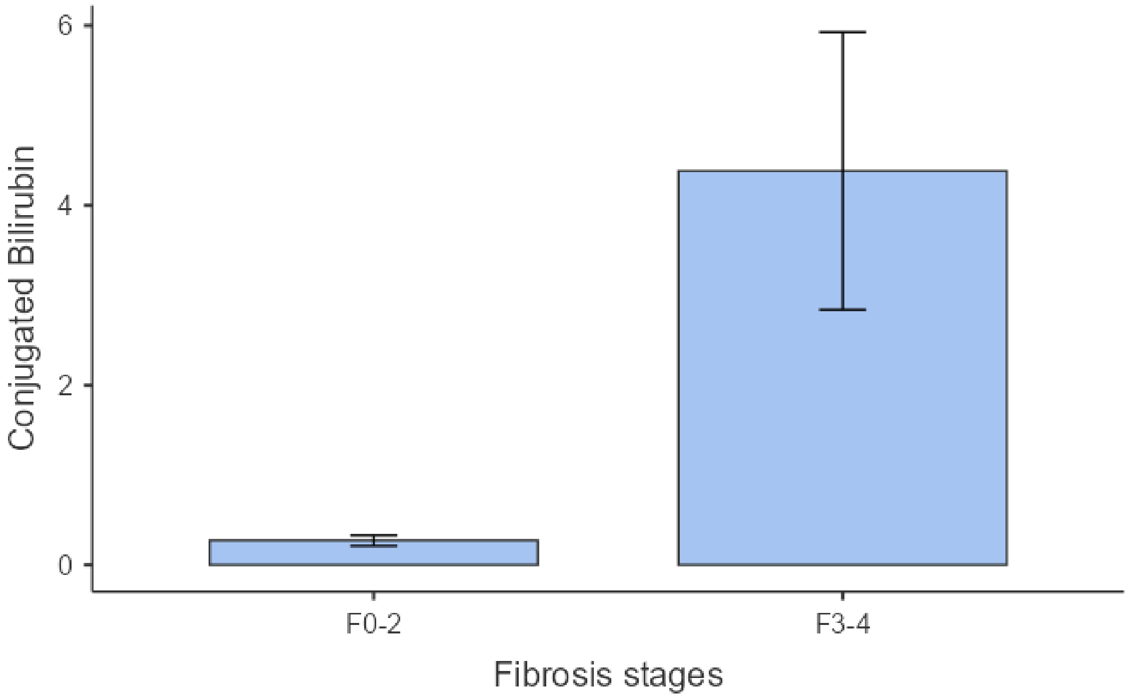

Supplementary Figure S1n - Albumin (g/dL)

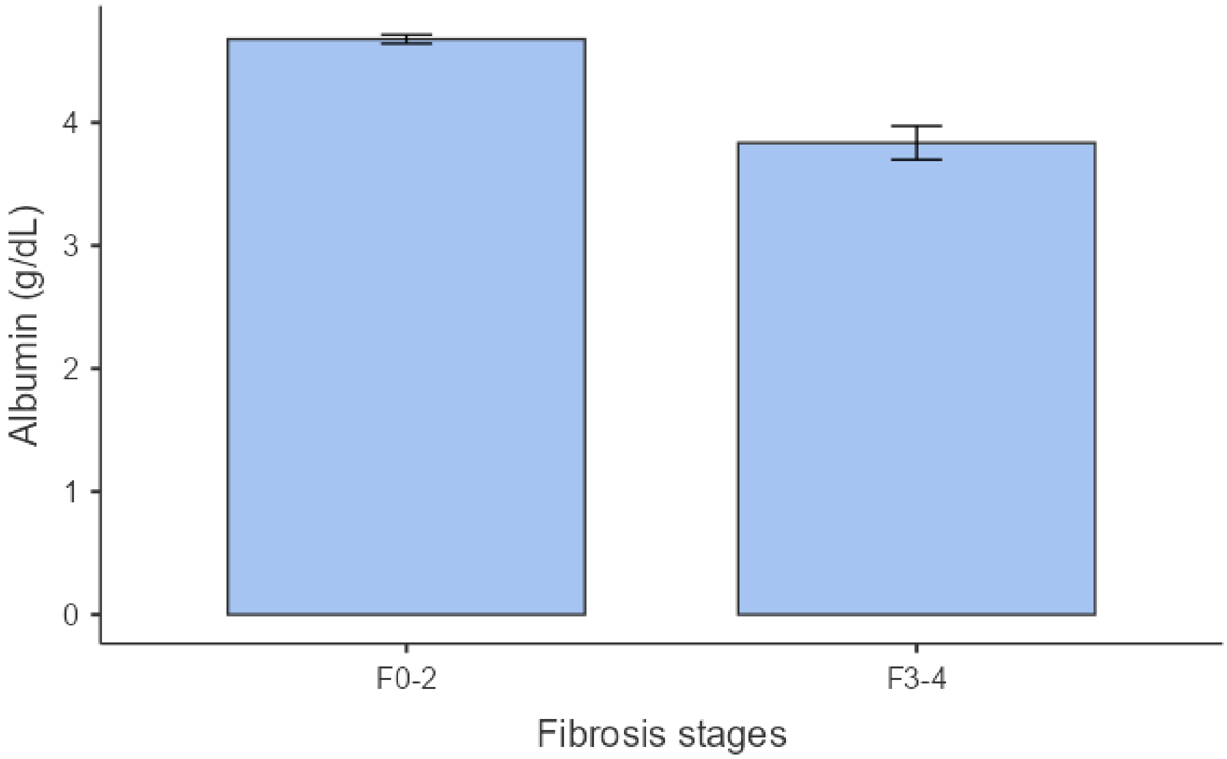

Supplementary Figure S1o - Cholesterol (mg/dL)

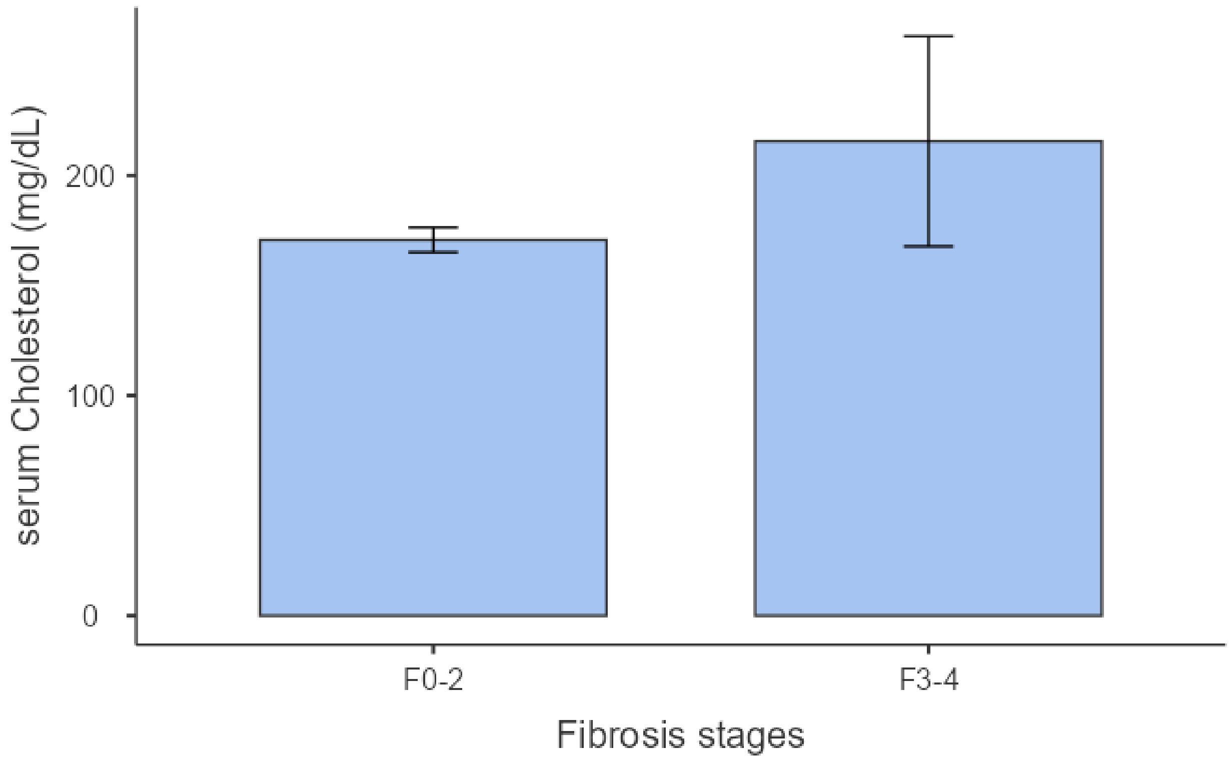

Supplementary Figure S1p - Triglycerides (mg/dL)

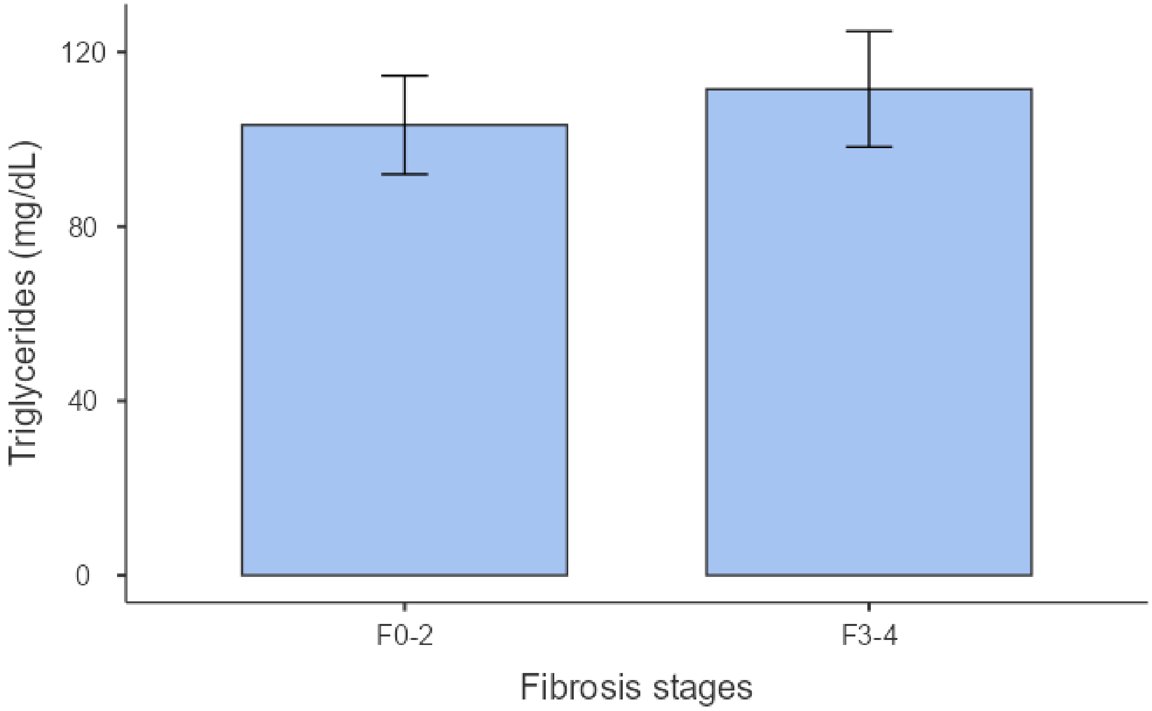

Supplementary Figure S1q - serum immunoglobulin G (IgG, mg/dL)

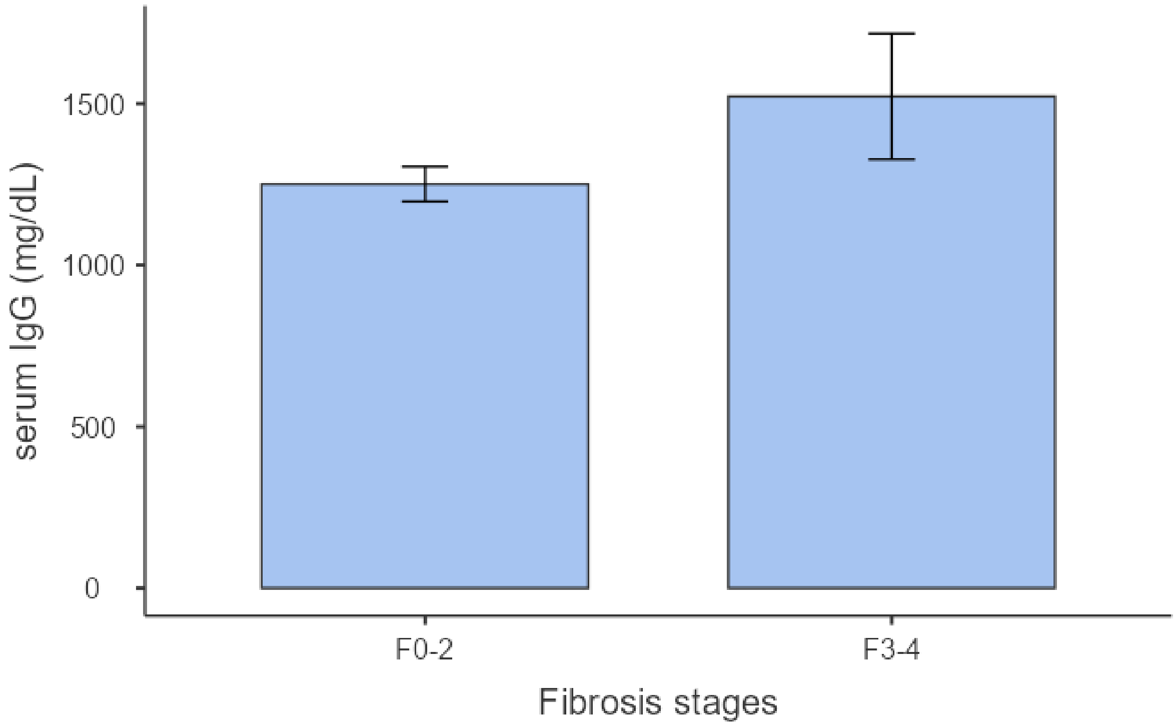

Supplement: Supplementary file 1 [file diagnostics-16-01102-s001.zip › Supplementary Figures S1 a-q bar plots for parameters compared between groups .pdf]
