# Supplementary material for: Performance of Blood-Based Indirect Scores Compared to Transient Elastography in Children with Chronic Liver Disease
Source: Diagnostics (Basel). 2026 Apr 6;16(7):1102. doi: 10.3390/diagnostics16071102 (PMC13074151; doi:10.3390/diagnostics16071102)

Supplementary Figures S2 a-i Scatterplots

Supplementary Figure S2a Scatterplot of kPa and AST/ALT values

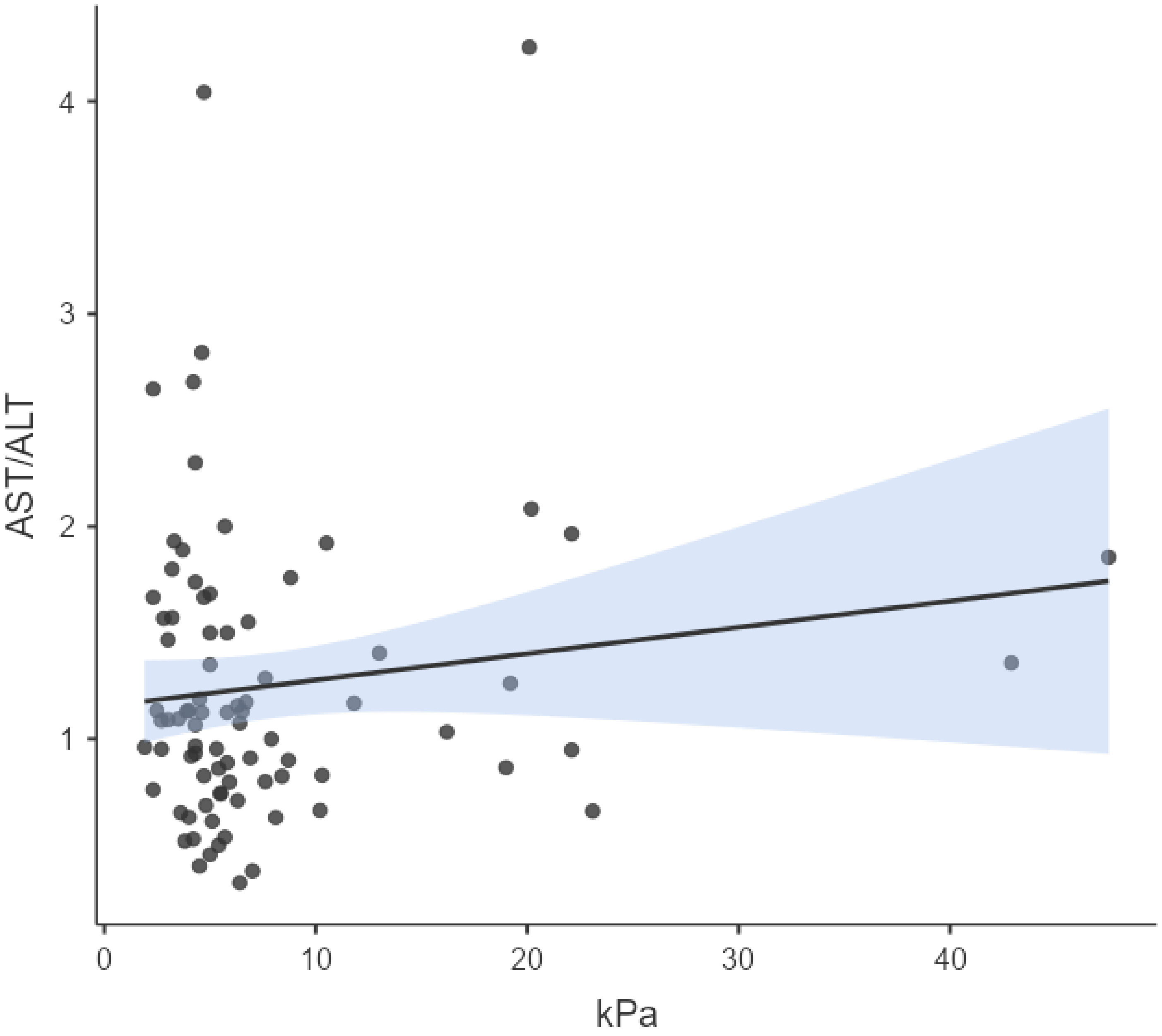

Supplementary Figure S2b Scatterplot of kPa and APRI score values

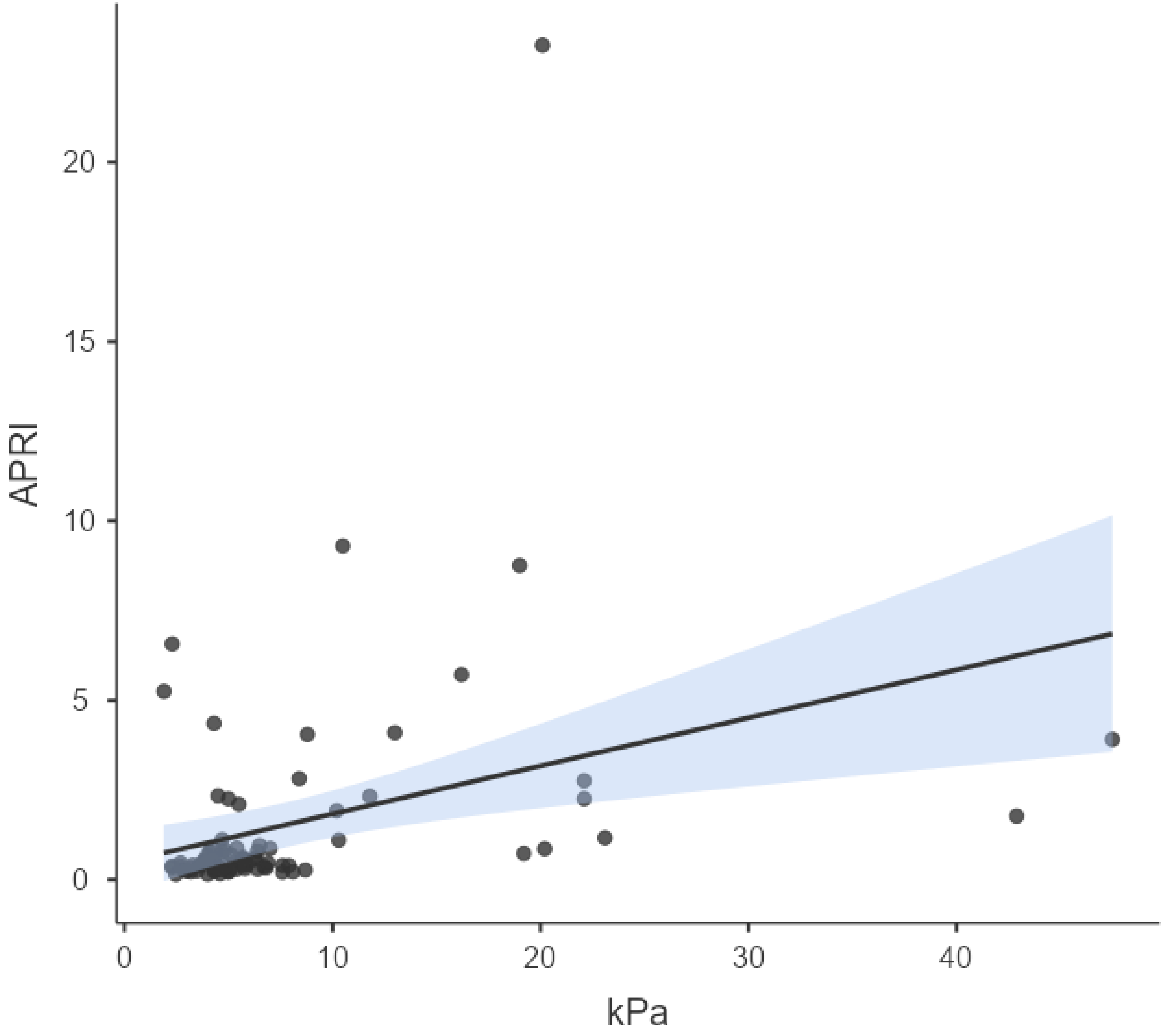

Supplementary Figure S2c Scatterplot of kPa and FibroIndex score values

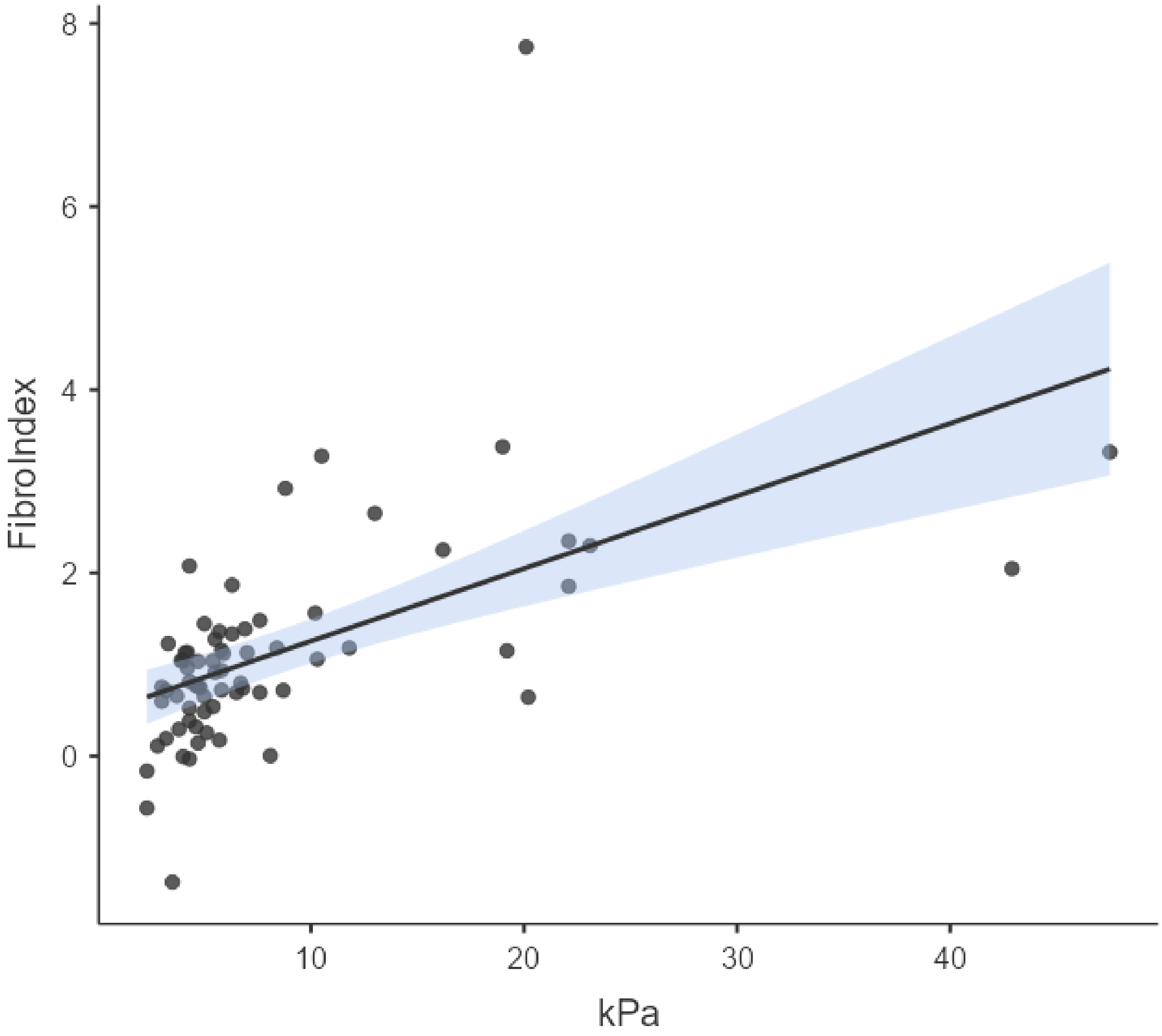

Supplementary Figure S2d Scatterplot of kPa and Forns score

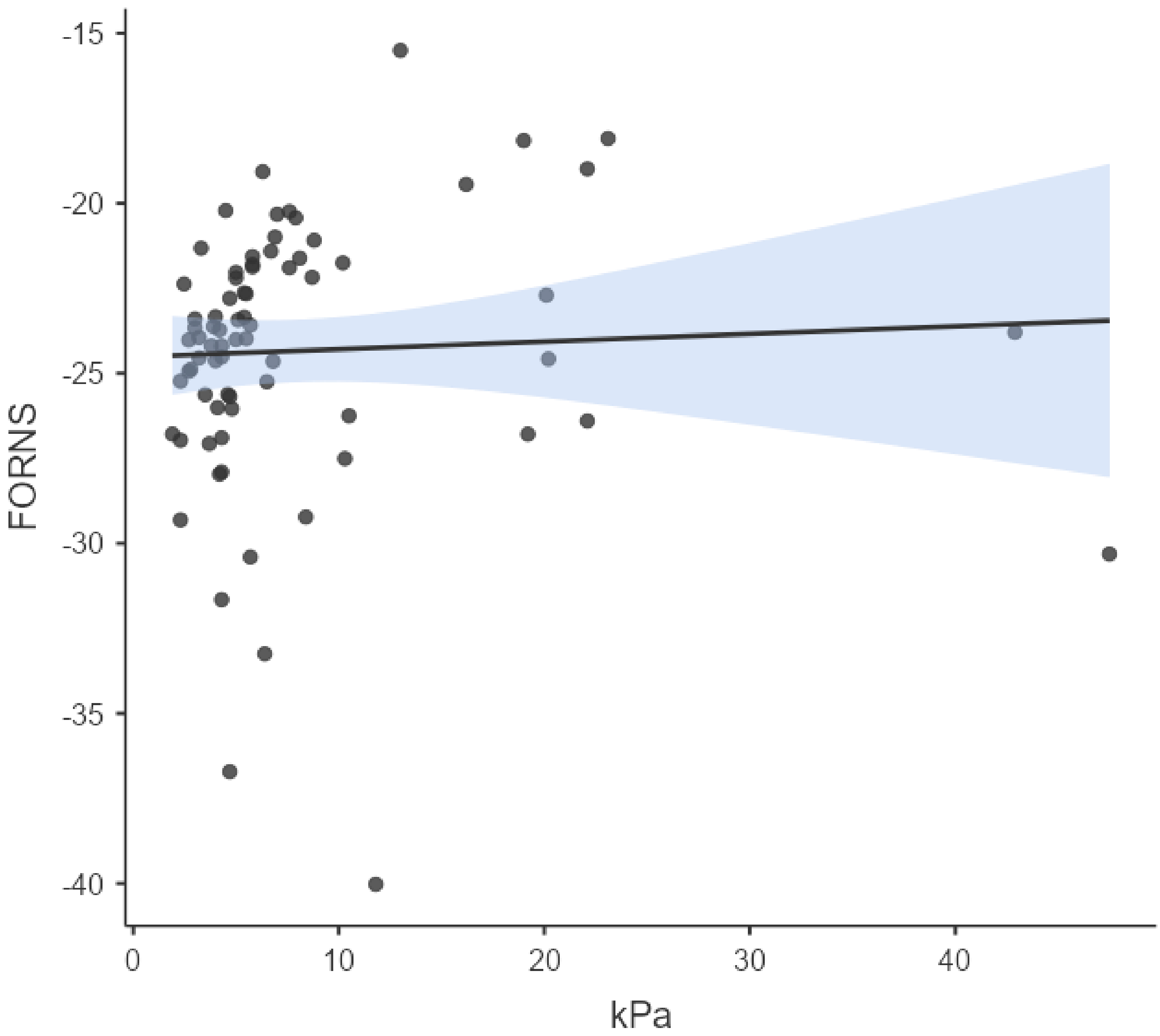

Supplementary Figure S2e Scatterplot of kPa and FIB4 score values

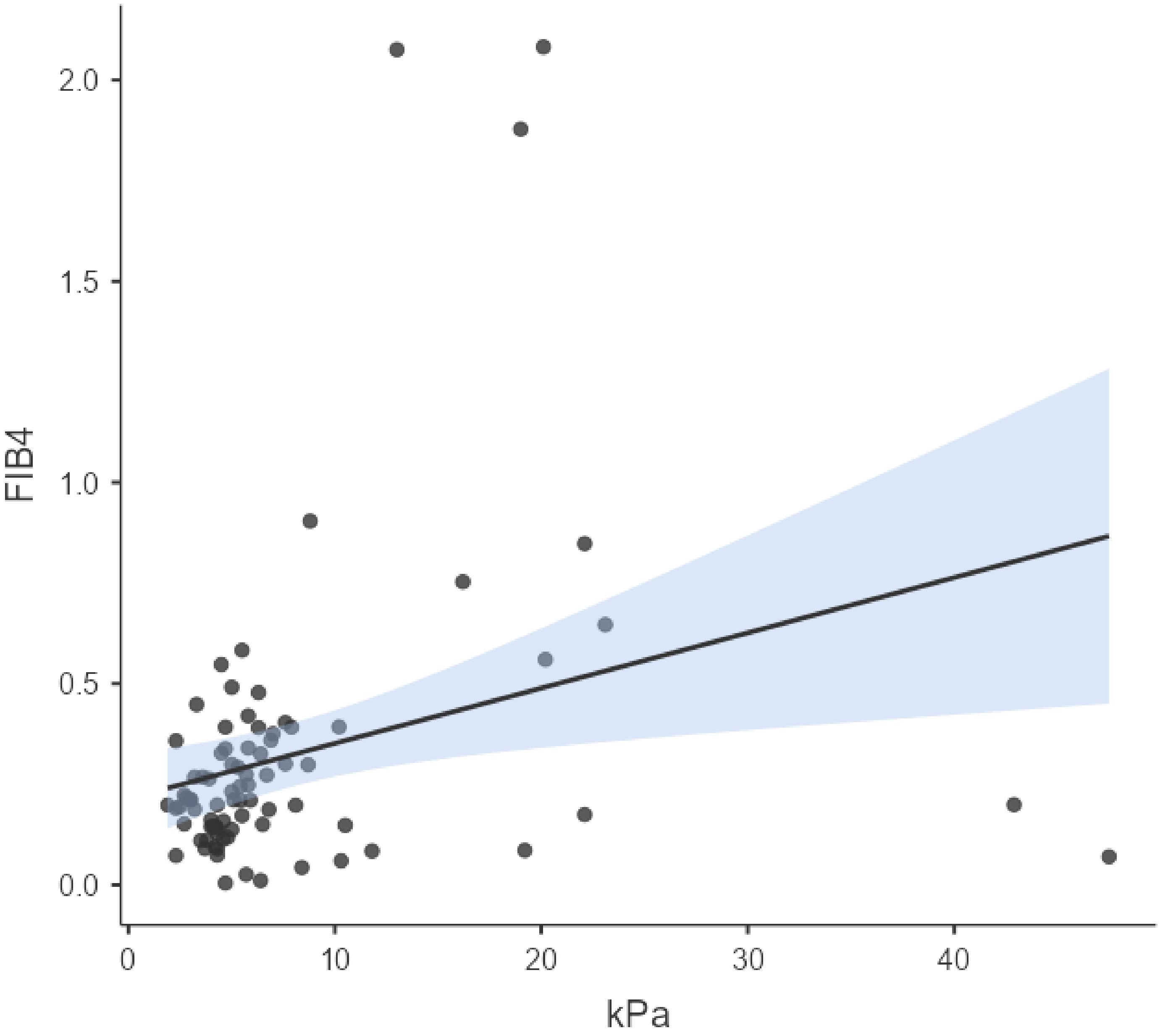

Supplementary Figure S2f Scatterplot of kPa and King’s score values

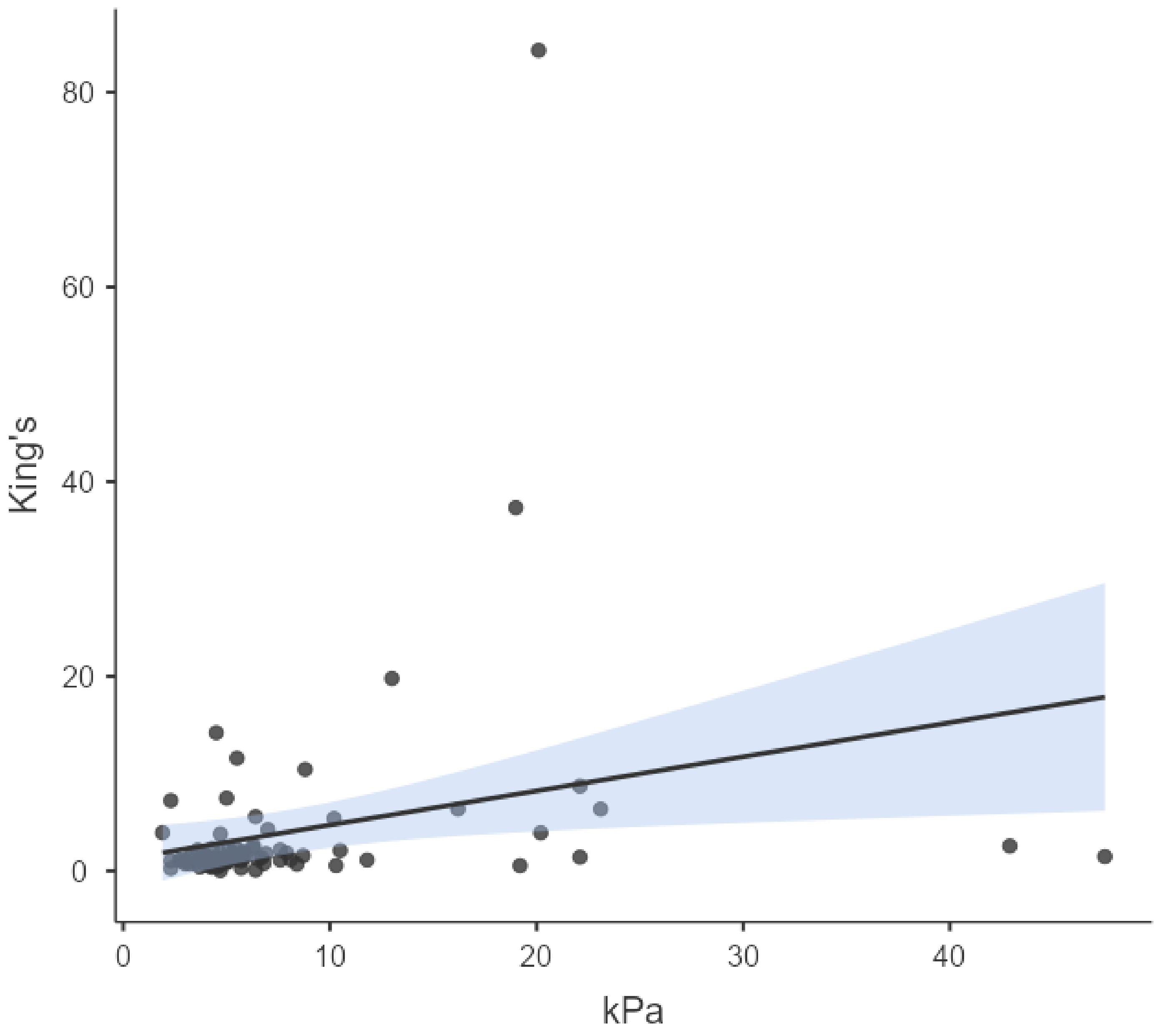

Supplementary Figure S2g Scatterplot of kPa and GPR score values

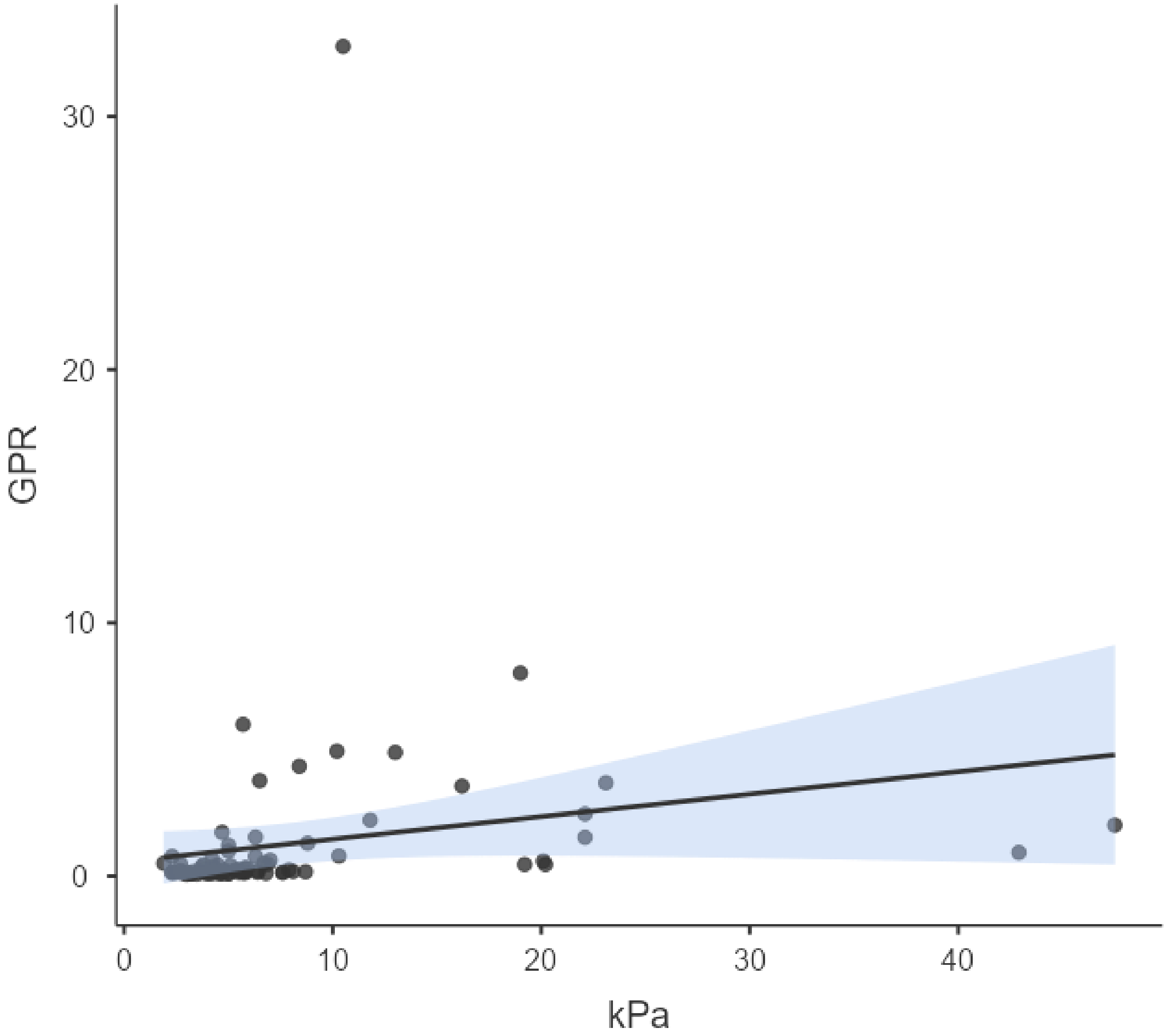

Supplementary Figure S2h Scatterplot of kPa and Lok’s Index values

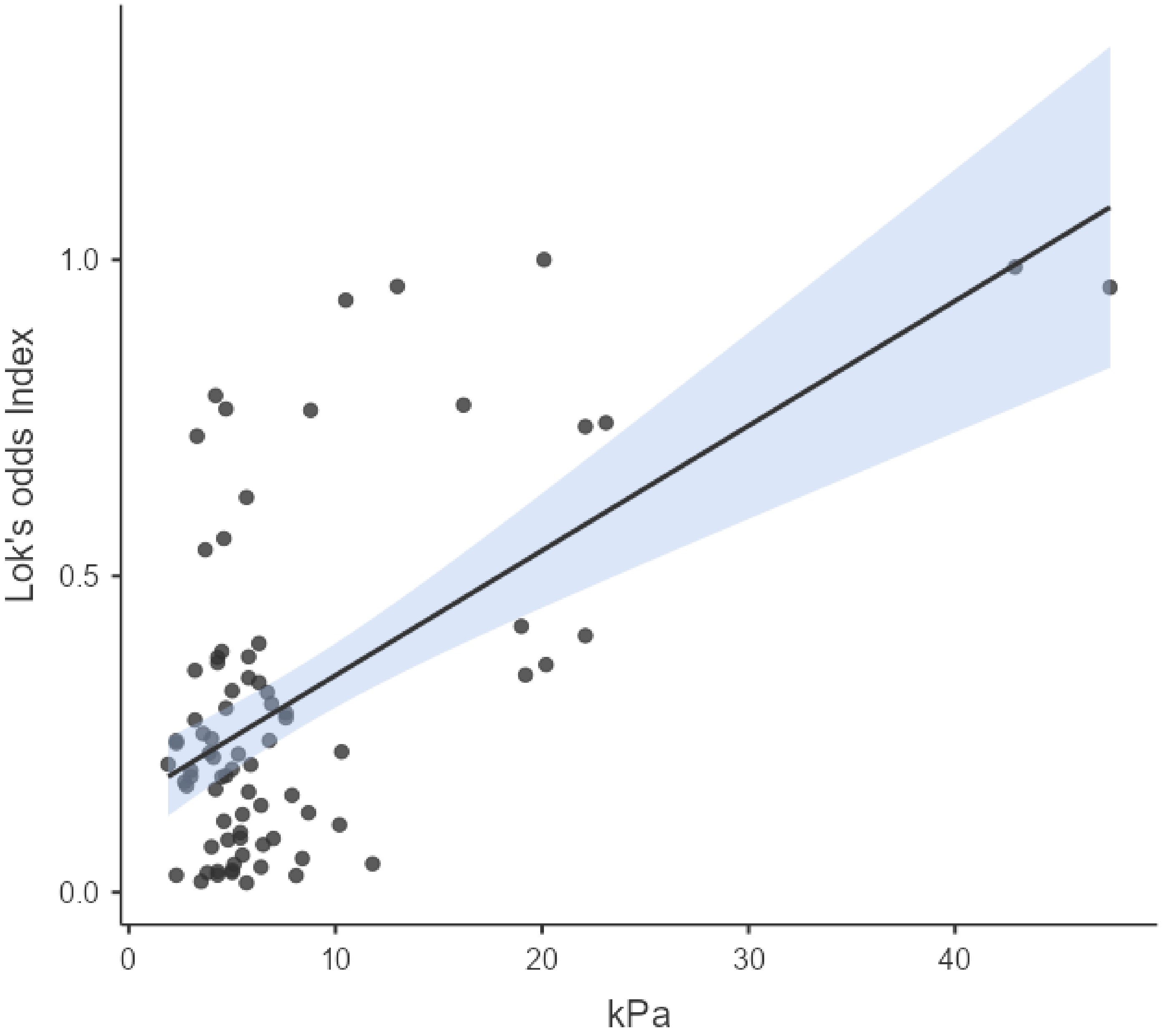

Supplementary Figure S2i Scatterplot of kPa and GUCI values

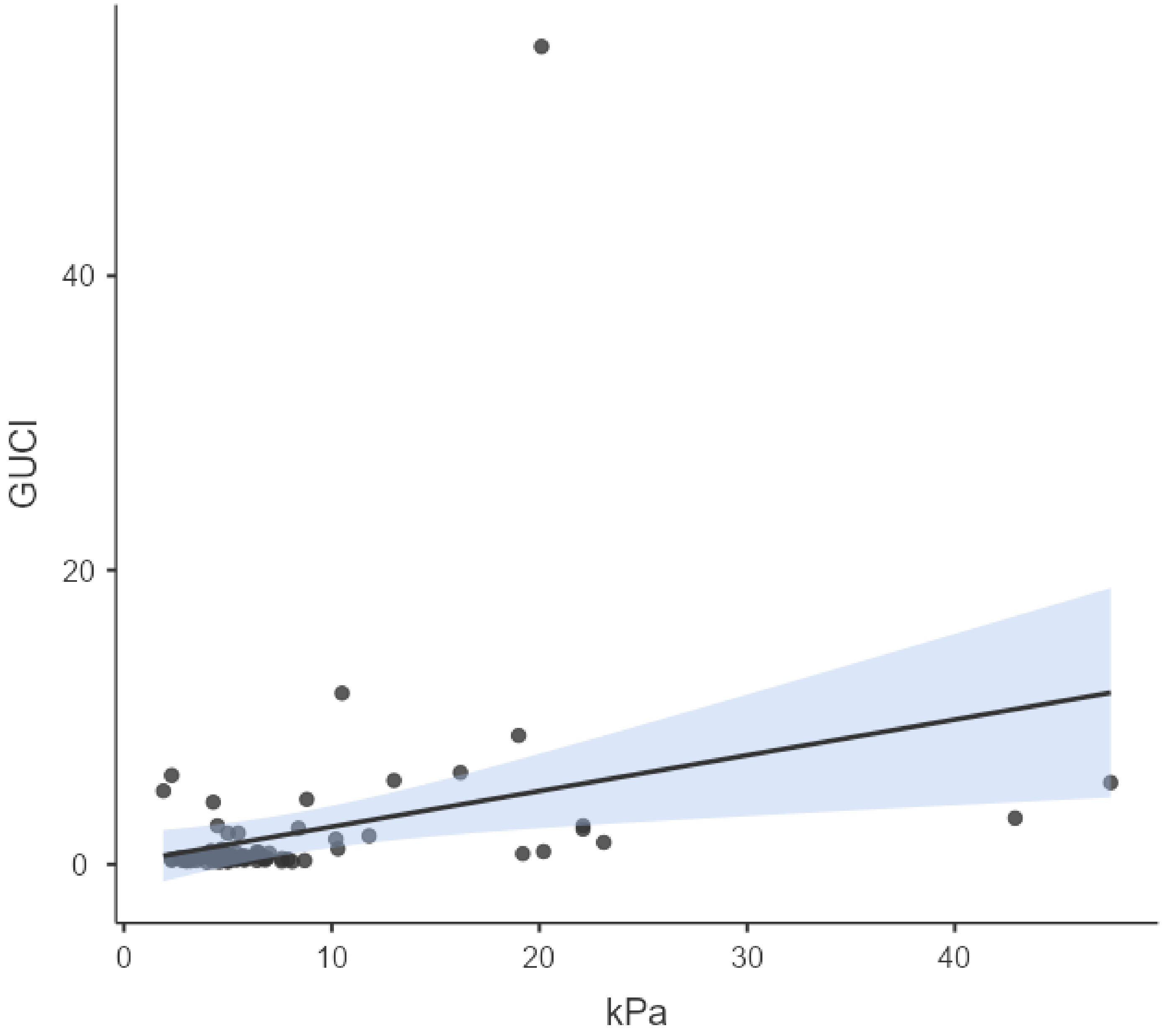

Supplement: Supplementary file 1 [file diagnostics-16-01102-s001.zip › Supplementary Figures S2a-i scatterplots.pdf]
