# Supplementary material for: Performance of Blood-Based Indirect Scores Compared to Transient Elastography in Children with Chronic Liver Disease
Source: Diagnostics (Basel). 2026 Apr 6;16(7):1102. doi: 10.3390/diagnostics16071102 (PMC13074151; doi:10.3390/diagnostics16071102)
Supplement: Supplementary file 1 [file diagnostics-16-01102-s001.zip › Supplementary Figures S3a-c ROC Curves.pdf]

Supplementary Fig S3a-c ROC curves

Supplementary Fig S3a ROC curves for the scores – discriminating between F0-1 and F2-4

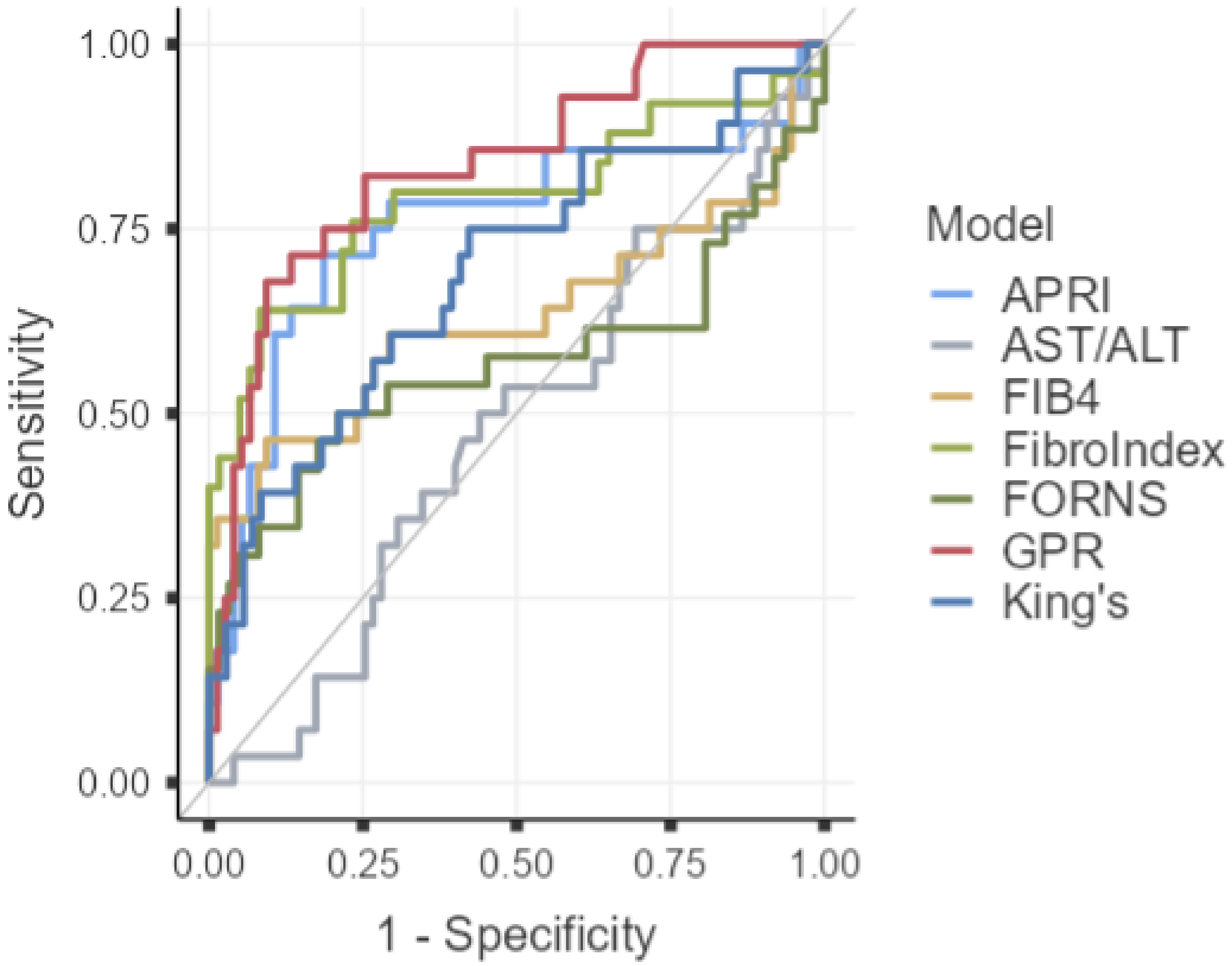

AST/ALT – AST-to-ALT ratio; APRI – AST to platelets ratio index; FIB4- FIB4 score; GPR – gamma glutamyl transferase to platelets ratio;

Supplementary Fig S3b ROC curves for the scores – discriminating between F0-2 and F3-4

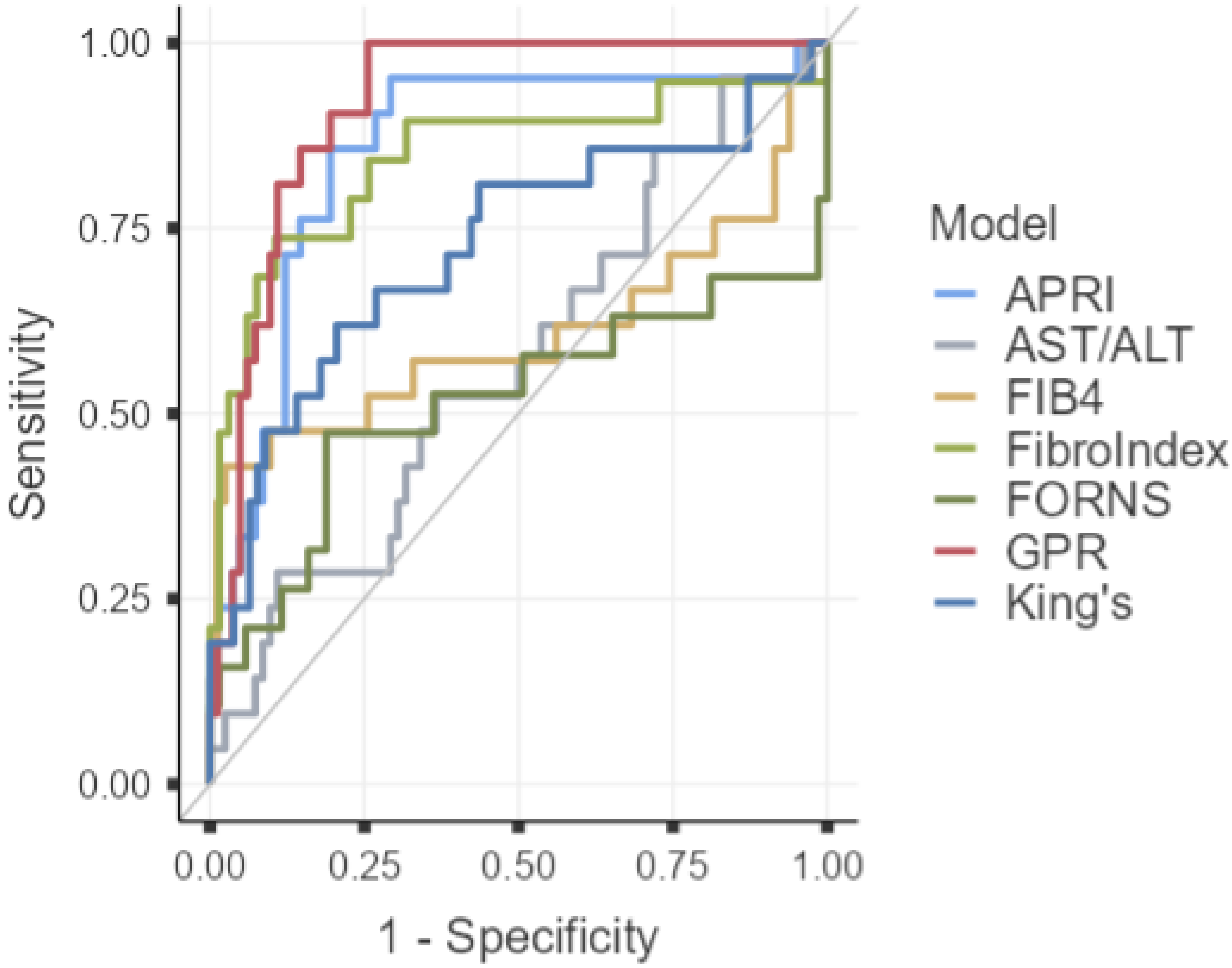

AST/ALT – AST-to-ALT ratio; APRI – AST to platelets ratio index; FIB4- FIB4 score; GPR – gamma glutamyl transferase to platelets ratio;

Supplementary Fig S3c ROC curves for the scores – discriminating between cirrhosis and all other stages of liver fibrosis

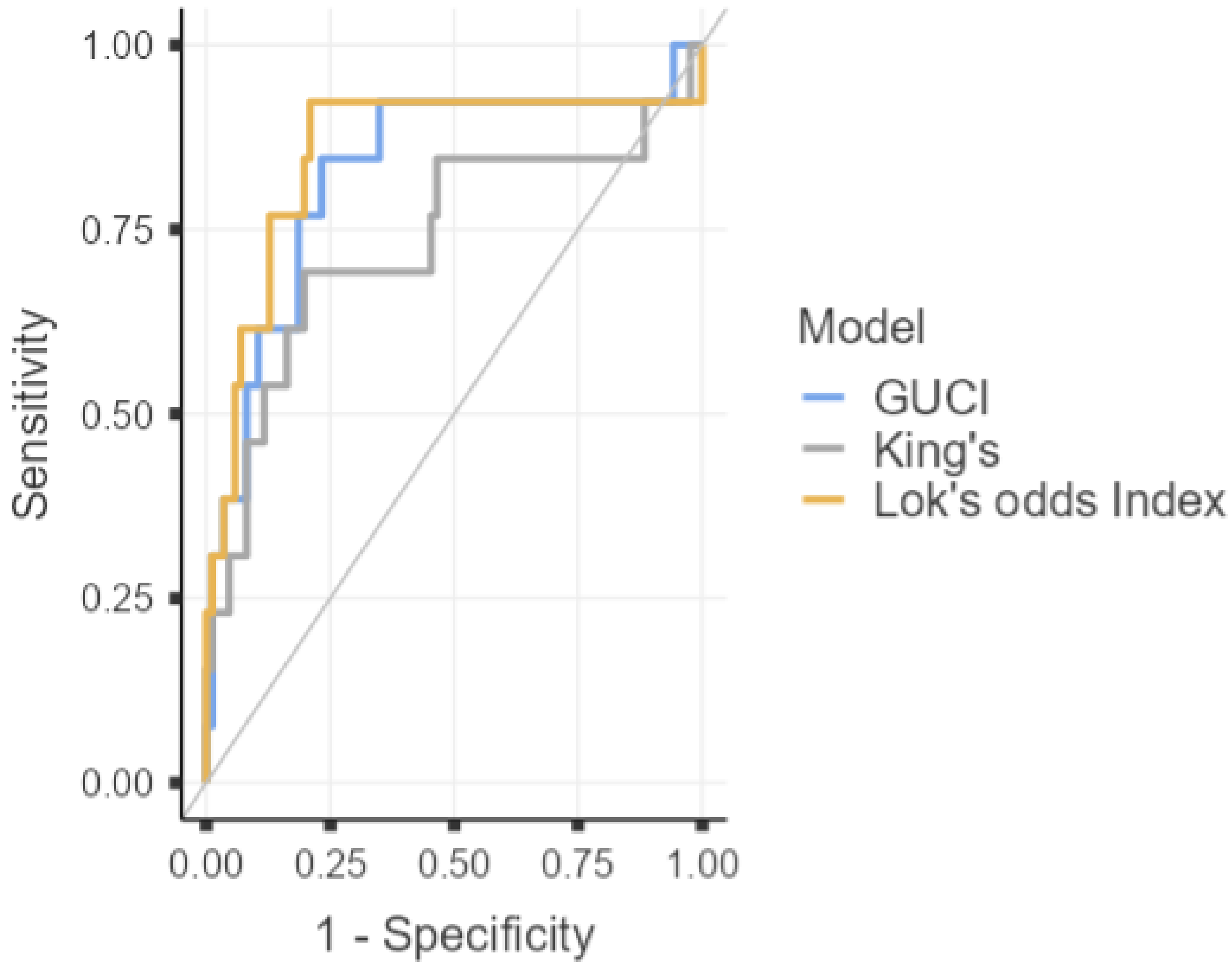

GUCI – Göteborg University Cirrhosis Index
